# Resveratrol for inflammatory bowel disease in preclinical studies: a systematic review and meta-analysis

**DOI:** 10.3389/fphar.2024.1411566

**Published:** 2024-06-14

**Authors:** Yuting Gu, Yijie Lou, Zhanyi Zhou, Xuan Zhao, Xiaolu Ye, Shuwen Wu, Haitao Li, Yunxi Ji

**Affiliations:** ^1^ The First School of Clinical Medicine, Zhejiang Chinese Medical University, Hangzhou, China; ^2^ Department of Acupuncture and Moxibustion, Zhejiang Provincial Hospital of Integrated Traditional Chinese and Western Medicine, Hangzhou, Zhenjiang, China; ^3^ Department of Digestive System, Jinhua Municipal Hospital of Traditional Chinese Medicine, Jinhua, China; ^4^ Department of General Practice, The First Affiliated Hospital of Zhejiang Chinese Medical University, Hangzhou, China

**Keywords:** resveratrol, inflammatory bowel disease, meta-analysis, systematic review, pharmacological mechanism

## Abstract

**Background:** Inflammatory bowel disease (IBD) is a chronic condition that can be managed with treatment, but it is challenging to get IBD cured. Resveratrol, a non-flavonoid polyphenolic organic compound derived from various plants, has a potential effect on IBD. The current research was set out to investigate the therapeutic effects of resveratrol on animal models of IBD.

**Methods:** A comprehensive search of PubMed, Embase, Web of Science, and Chinese databases was performed. The literature search process was completed independently by two people and reviewed by a third person. The risk of bias in the included literature was assessed using the Collaborative Approach to Meta Analysis and Review of Animal Data from Experimental Stroke (CAMARADES) 10-point quality checklist. The meta-analysis utilized Review Manager 5.4 software to evaluate the efficacy of resveratrol, with histopathological index as the primary outcome measure. Subgroup analysis was conducted based on this indicator. Additionally, meta-analyses were carried out on different outcomes reported in the literature, including final disease activity index, final body weight change, colon length, splenic index, and inflammatory factors.

**Results:** After conducting a thorough literature search and selection process, a total of 28 studies were ultimately included in the analysis. It was found that over half of the selected studies had more than five items with low risk of bias in the bias risk assessment. Relevant datas from included literature indicated that the histopathological index of the resveratrol group was significantly lower than that of the control group (WMD = −2.58 [-3.29, −1.87]). Subgroup analysis revealed that higher doses of resveratrol (>80 mg/kg) had a better efficacy (WMD = −3.47 [-4.97, −1.98]). Furthermore, The data summary and quantitative analysis results of SI and colon length also showed that resveratrol was effective in alleviating intestinal mucosal pathological injury of IBD. In terms of biochemical indicators, the summary analysis revealed that resveratrol affected interleukin-1β (IL-1β), interleukin-6 (IL-6), interleukin-8 (IL-8), interleukin-10 (IL-10), tumor necrosis factor-α (TNF-α), transforming growth factor-β (TGF-β), interferon-γ (IFN-γ), malondialdehyde (MDA), myeloperoxidase (MPO), superoxide dismutase (SOD), and prostaglandin E2 (PGE2) significantly. These effects may be attributed to the mechanism of resveratrol in regulating immune response and inhibiting oxidative stress.

**Conclusion:** This review suggests that resveratrol demonstrated a notable therapeutic impact in preclinical models of IBD, particularly at doses exceeding 80 mg/kg. This efficacy is attributed to the protective mechanisms targeting the intestinal mucosa involved in the pathogenesis of IBD through various pathways. As a result, resveratrol holds promising prospects for potential clinical use in the future.

## 1 Introduction

Inflammatory bowel disease (IBD) is a chronic, non-specific inflammatory condition that affects the bowel and mainly includes two types, namely, ulcerative colitis (UC) and Crohn’s disease (CD) (Lee and Eun, 2022). A systematic review of the global IBD epidemic reveals that Europe and North America have the highest incidence of IBD. In many countries in North America, Oceania, and Europe, the incidence of IBD exceeds 0.3% (Ng et al., 2017). On the other hand, newly industrialized countries in Africa, Asia, and South America have seen an increase in IBD incidence since 1990 (Ng et al., 2017; Mak et al., 2020; Agrawal and Jess, 2022; Aniwan et al., 2022). The IBD has a complex pathophysiology, and it has detrimental effects on the body. Its main clinical symptoms include abdominal pain, diarrhea, mucus, pus, and bloody stools. Initial prolonged damage to the intestinal lining can impair nutrient absorption (Seyedian et al., 2019). Repeated attacks of the disease may lead to the development of intestinal fistulas. As a result, there is a risk of leakage of digestive juices and feces, which can cause infection and pain, and this complication is particularly severe in individuals with CD (Seyedian et al., 2019). In addition, in severe cases, complications such as intestinal perforation, acute bleeding, and intestinal obstruction may arise, necessitating emergency surgery and contributing to a poor prognosis. Numerous studies have shown that compared with the general population, IBD patients have a higher risk of developing cancer, especially colorectal cancer (CRC), and additionally, the prognosis of IBD-related CRC is worse than that of sporadic CRC (Mattar et al., 2011). This can be attributed to the fact that the chronic inflammatory state caused by IBD promotes mucosal proliferation and does not follow the traditional adenoma-carcinoma sequence (Keller et al., 2019; Sato et al., 2023). In addition, IBD can impose a significant economic burden on patients, often affecting their treatment adherence.

During the process of IBD, the physical barrier of the intestinal mucosa can deteriorate, consequently affecting both innate and adaptive immunity (Beisner et al., 2010; Piechota-Polanczyk and Fichna, 2014). Activation of innate immune cells, such as macrophages, can result in the production of superoxide and nitric oxide by nitrogen oxide (NOX) and nitric oxide synthase (NOS), in addition to the generation of oxidant peroxynitrite (Mangerich et al., 2012; Piechota-Polanczyk and Fichna, 2014; Guan, 2019). Furthermore, other immune cells also contribute to the production of reactive oxygen species (ROS) during metabolic processes, leading to significant tissue damage (Piechota-Polanczyk and Fichna, 2014; Guan, 2019). Simultaneously, the imbalance between helper T cells (Th) and regulatory T cells are essential to the progression of IBD (Uniken et al., 2017; Guan, 2019; Qiuping et al., 2021; Korta et al., 2023). The CD is primarily mediated by Th1 cells, while UC inflammation is mainly caused by Th2 cells (Uniken et al., 2017). The interleukin 23/T helper cell 17 (IL-23/Th17) pathway also affects the progression of IBD (Toussirot, 2012; Guan, 2019; Qiuping et al., 2021; Korta et al., 2023). Additionally, the activation of nuclear factor κB (NF-κB) and other signaling pathways results in the uncontrolled release of inflammatory cytokines during the IBD process and contributes to the inflammatory cascade reaction, the proliferation of memory T cells, and alterations in the intestinal microenvironment, ultimately resulting in the persistent development of IBD (Piechota-Polanczyk and Fichna, 2014; Guan, 2019; Shen et al., 2020; Zhang Z. et al., 2022; Ni et al., 2022).

Currently, a combination of various methods is used in the treatment of IBD, including 5-aminosalicylate (5-ASA), thiopurines, anti-tumor necrosis factor drugs, probiotics, antibiotics, and surgery (Luo et al., 2022). However, these treatment options have certain limitations. Conventional treatment often involves the use of immunosuppressive and anti-inflammatory drugs, which can lead to serious side effects and complications. Biologic therapy, while effective, can vary greatly in its effectiveness among individuals and is often expensive, impacting patient compliance (Shivaji et al., 2020). Many patients demonstrate both primary and secondary drug resistance, and adjusting the biological treatment plan following the detection of resistance can lead to an increase in the patient’s side effects and medical costs (Zhou WP. et al., 2021). Furthermore, even if the initial treatment effectively manages the condition, a significant number of patients still encounter disease recurrence, which poses a challenge when it comes to adjusting treatment strategies.

Extracting effective compounds from plants and herbs for complementary and adjunctive treatment is a crucial avenue for future treatment of IBD. Resveratrol is a natural polyphenolic compound that can be derived from various sources such as cassia seed, mulberry bark, and tea (Zhang et al., 2021). Numerous studies have demonstrated the diverse biological functions of resveratrol, including metabolic regulation, anti-inflammatory effects, antioxidant properties, anti-cancer activity, anti-aging effects, and improvement of renal function in diabetic patients (Breuss et al., 2019; Huang et al., 2020; Zhou DD. et al., 2021; Ren et al., 2021; Yang et al., 2021). Based on the pathological characteristics of IBD, resveratrol may inhibit oxidative stress and inflammation to alleviate the severity of IBD (Yao et al., 2023). Currently, there are limited clinical RCT studies on the therapeutic effect of resveratrol on IBD. The present study aimed to address these shortcomings with a meta-analysis of all relevant studies, to provide more convincing and scientific evidence for the clinical application of resveratrol in the treatment of IBD.

## 2 Methods

### 2.1 Study selection

Animal experimental studies that investigate the effects of resveratrol in the treatment of IBD were systematically searched in PubMed, Embase, Web of Science, China Knowledge Infrastructure Network, and Wanfang database electronic database. To ensure comprehensive coverage of the literature, we conducted searches in OpenGrey, the National Technical Information Service, Health Canada, and CADTH-Canadian Agency for Drugs and Technologies. Despite extensive searching in large health databases, no studies on resveratrol for IBD treatment were identified. The time span for literature search was from inception to 23 September 2023, without any language restrictions. MeSH terms used in our research process included “inflammatory bowel disease,” “colitis, ulcerative,” “IBD,” “ulcerative colitis,” “colitis,” “anti-colitis,” “Crohn disease,” “Crohn’s disease,” “Crohns disease,” “Crohn’s enteritis,” “resveratrol,” “3,5,4-trihydroxystilbene,” “trihydroxystilbene,” “SRT 501,” “resveratrol-3-sulfate,” “cis-resveratrol,” “trans-resveratrol,” “trans-resveratrol-3-O-sulfate,” and “3,4,5-stilbenetriol.”

The literature was screened by two reviewers (Y.Y.G and Y.J.L) based on the abstracts and full texts of the research obtained. Any discrepancies were resolved through discussion with a third reviewer (Y.X.J).

### 2.2 Eligibility criteria

#### 2.2.1 Types of studies

Animal studies investigating the therapeutic effects of resveratrol in rat or mouse models with IBD were included. Clinical case reports or *in vitro* studies were excluded.

#### 2.2.2 Animal models

There were no strict restrictions on sex, age, or strain of rats or mice induced with IBD. Tritrobenzene sulfonic acid (TNBS), dextran sodium sulfate (DSS), and oxazolone (OXZ) were used to induce IBD in rats or mice. However, models created using radiation, allergic enteritis models induced by ovalbumin, and genetically deficient mouse models of spontaneous chronic colitis that lack the anti-inflammatory cytokine interleukin-10 (IL-10−/− mouse) were excluded. The three models exhibited significant heterogeneity in pathogenesis compared to the included IBD models. The genetic defect in the IL-10−/− model results in the loss of IL-10-mediated inhibition of macrophage and T cell function, preventing antigen-stimulated immune regulation (Kühn et al., 1993). As a result, it exhibits progressive development of chronic enteritis without intervention, contrasting with the chemical-induced IBD model in healthy animals. Additionally, the modeling cycle for this model is notably longer than the included model (Singh et al., 2012; Holcomb et al., 2023). OVA primarily induces allergic enteritis, predominantly mediated by type I hypersensitivity through IgE (Kato et al., 2023). Radiation-induced models mainly focus on radiation enteritis (Sun et al., 2020). Detailed characterizations of the relevant exclusion literatures were provided in the Supplementary Table S1.

#### 2.2.3 Interventions

The treatment group received resveratrol with no limitations on the dose, dosage form, route, and time of administration. The control group either remained untreated or was treated with vehicles. Studies that combined resveratrol with other interventions for the treatment group were excluded. Studies without a control group were also excluded.

### 2.3 Outcome measures

#### 2.3.1 Primary outcome

The histopathological index, extracted from animal experimental literature, serves as the primary outcome for quantitative and subgroup analysis. This index is a valuable tool for measuring intestinal mucosal damage in IBD.

#### 2.3.2 Secondary outcomes

Secondary outcome measures extracted from the literature for meta-analysis included final DAI score, final body weight change, spleen index, colon length, inflammatory factor levels, oxidative stress-related biochemical indices, and enzyme metabolites.

### 2.4 Literature selection and data extraction

Two authors independently extracted data from the included studies. These data included title, first author, year of publication, animal strain, weight and sex, number of animals in each group, method used to induce IBD, resveratrol administration (including dose, method, and time), and measurable outcome measures. The mean and standard deviation were extracted to compare the values of each variable. Following the recommended intervention review in the Cochrane Systematic Manual, the experimental group was combined to create a single-pair comparison for study groups with multiple interventions. We used GetData graph digitizer 2.24 to interpret the graph data. Any differences were resolved through discussion with a third reviewer (Y.X.J).

### 2.5 Assessment of risk of bias in the included studies

The quality and design of all included studies were assessed using the ten-point quality checklist published by Collaborative Approach to Meta Analysis and Review of Animal Data from Experimental Studies (CAMARADES) (Sena et al., 2007). Ratings of “yes,” “no,” or “unclear” for the criteria items indicate low risk, high risk, or an inadequate assessment of risk of bias, respectively. The evaluation process was conducted independently by two individuals (Y.Y.G and Y.J.L), and in the event of disagreements, the opinion of a third person was sought (Y.X.J).

### 2.6 Statistical analysis

RevMan 5.4 software was used for summary analysis of data included in the study. Mean difference (MD) was used for the same unit, while standardized MD was used for different units. Cochrane I^2^ score was used to determine heterogeneity between groups. Heterogeneity was assumed if the *p*-value of the Chi-square test was less than 0.10. The I^2^ value greater than 50% indicated high heterogeneity. The fixed effects model was used for homogeneous clinical and statistical analysis, while the random effects model was used for heterogeneous clinical and statistical analysis. A subgroup meta-analysis was conducted by three dose levels of resveratrol: high-dose (>80 mg/kg), medium-dose (>40 mg/kg, ≤80 mg/kg), and low-dose (≤40 mg/kg). At the same time, subgroup analysis was performed based on the gender and model of the experimental animals, the concentration and duration of the modeling reagent, the mode of resveratrol administration. Line graphs were generated using GraphPad Prism 10 software to show trends in DAI values between the two groups.

## 3 Results

### 3.1 Description of the included studies

**FIGURE 1:**
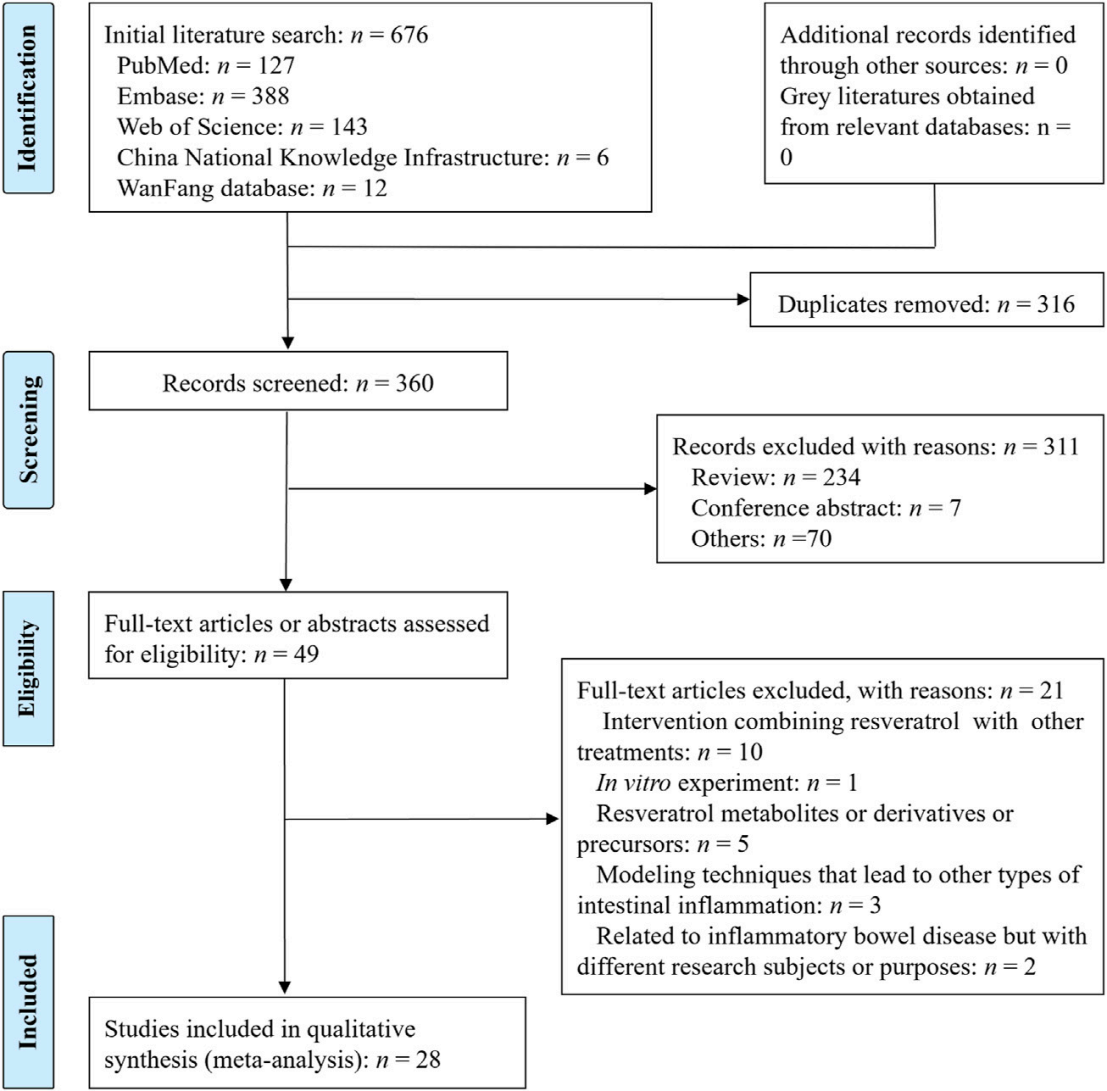
Flow diagram of studies included in this review.

The process of literature screening is shown in Figure 1 (Liberati et al., 2009). A total of 676 articles were retrieved using specific search strategies. After excluding 316 duplicates, we further excluded 234 reviews, 7 conference abstracts, and 70 other records that were unrelated to the research topic. We obtained 49 articles for full-text review. Finally, 28 articles were included in this study. Among the excluded articles, 10 focused on the combination of resveratrol with other therapeutic methods as the intervention factor (Youn et al., 2009; Lozano-Pérez et al., 2014; Liu et al., 2018; Seoane-Viaño et al., 2019; Zhang et al., 2019; Gandhi et al., 2020; Lize et al., 2020; Naserifar et al., 2020; Pujara et al., 2021; Li et al., 2023), 1 involved an *in vitro* experiment (Sabzevary-Ghahfarokhi et al., 2020), 5 primarily studied resveratrol metabolites, derivatives, and precursors (Larrosa et al., 2010; Chen et al., 2017; Zhang B. et al., 2022; Fei et al., 2022; Zhang et al., 2023), 3 used modeling techniques to induce other types of intestinal inflammation (Singh et al., 2012; Sun et al., 2020; Bilotta et al., 2022), and 2 were related to IBD but had different research subjects or purposes (Wagnerova et al., 2017; Lu et al., 2019). Among the included studies, 19 were published in English and 9 were in Chinese (Jun et al., 2010a; Jun et al., 2010b; Singh et al., 2012; Wagnerova et al., 2017; Lu et al., 2019; Shenggao et al., 2019; Jianheng, 2021; Jianheng et al., 2021; Qiuping et al., 2021).

### 3.2 Characteristics of the included studies

The features of the included research are summarized in Table 1 (Martín et al., 2004; Larrosa et al., 2009; Jun et al., 2010a; Jun et al., 2010b; Cui et al., 2010; Singh et al., 2010; Yao et al., 2010; Yao et al., 2011; Abdin, 2013; Yao et al., 2015; Yildiz et al., 2015; Mayangsari and Suzuki, 2018; Alrafas et al., 2019; Qiuping et al., 2019; Shenggao et al., 2019; Xin et al., 2019; Alrafas et al., 2020; Li et al., 2020; Pan et al., 2020; Wang et al., 2020; Jianheng, 2021; Jianheng et al., 2021; Li et al., 2021; Qiuping et al., 2021; Zhu et al., 2021; Liujing et al., 2023; Xu et al., 2023; Zhou et al., 2023). Among the studies included, 23 studies were conducted in mice, and 5 studies were conducted in rats. In terms of modeling techniques, 22 studies utilized oral DSS induction modeling, 5 studies employed TNBS intrarectal administration to induce IBD, and 1 study utilized the OXZ induction model. Among the studies using DSS molding reagents, the application time of reagents in a cycle varied from 4 to 10 days, with most studies applying them for 7 days, and 16 studies employed 1 cycle modeling, 3 studies employed 2 cycle modeling, 1 study employed 2.5 cycle modeling, while 2 studies employed 4 cycle modeling. Generally, the molding cycle was shorter. Among the studies using TNBS modeling reagent, 4 studies used TNBS mixed with ethanol, and 1 study used DMSO as the carrier. The sample sizes of all included studies ranged from 15 to 90.

**TABLE 1:** Characteristics of the included studies.

Study	Animal	Model		Sample size	Groups	Dosage of RSV	Administration method of RSV	Assessment time	Outcomes
Oral administration by dissolving in water									
Martín AR 2004	Male Wistar rats, 180–220 g		10 mg TNBS in 0.25 mL 50% ethanol into colon through cannula	11/15/14/12/12	A. Normal saline control B. Ethanol group C. Model control D. TNBS + Low-dose RSV E. TNBS + High-dose RSV	5 mg/kg/day and 10 mg/kg/day	RSV in 0.9% saline solution was gavaged 48, 24 and 1 h prior to the induction of colitis and 24 h later	—	Macroscopic damage score, weight loss, weight/length of rat colon, diarrhoea, adhesions, HE staining, MPO, IL-1β, PGE_2_, PGD_2_, cellular distribution of COX-1/COX-2
Wang JH 2021a	Male BALB/c mice, 7–8 weeks, 20–22 g		3% DSS for 7 days, 14 days a cycle, 1 cycle	15/15/15/15/15/15	A. Normal control B. Model control C.DSS + Low-dose RSV D.DSS + Moderate-dose RSV E.DSS + High-dose RSV F.DSS + SASP (500 mg/kg/day)	10 mg/kg/day, 50 mg/kg/day and 100 mg/kg/day	RSV was gavaged daily for 14 days after modeling	—	DAI, HE stainin, IL-10 TNF-α, IL-1β, IL-6, TLR4/MYD88/NF-κB P65
Wang JH 2021b	Male BALB/c mice, 7–8 weeks, 20–22 g		3.5% DSS for 7 days, 14 days a cycle, 1 cycle	15/15/15/15/15/15	A. Normal control B. Model control C. RSV (100 mg/kg/day) D.DSS + Low-dose RSV E.DSS + Moderate-dose RSV F.DSS + High-dose RSV	10 mg/kg/day, 50 mg/kg/day and 100 mg/kg/day	RSV was gavaged 3 times daily for 14 days after modeling	1-15d	Weight, general condition, DAI, colon elasticity/length, HE staining, IL-10 TNF-α, IL-1β, IL-6, SIRT1, mTOR, Atg12, Beclin-1, LC3, Atg12, CD4+T/CD8+T
Singh UP 2010	Female C57BL/6 mice, 8–12 weeks		3% DSS for 7 days, 14 days a cycle, 1 cycle	6/6/6/6/6/6	A. Normal control B. RSV C. DSS + Low-dose RSV D. DSS + Moderate-dose RSV E. DSS + High-dose RSV D. DSS + vehicle	10 mg/kg/day, 50 mg/kg/day and 100 mg/kg/day	RSV was gavaged daily for 14 days after modeling	1-14d	Weight loss, HE staining, SAA, IL-6, IL-1β, IFN-γ, TNF-α, inflammation scores, colon length, CD4^+^ T cells with IFN-γ/TNF-α, macrophages/T cells percentage, COX1/2, SIRT1, p-IκBα
Zhu F 2021	Male BALB/c mice, 6–8 weeks	5% DSS, 7 days, 14 days a cycle, 1 cycle		5/5/5/5	A. Normal control B. Model control C. DSS + RSV D.DSS + sulfasalazine (200 mg/kg)	100 mg/kg	RSV was gavaged daily from 1 to 7days after modeling	0, 3, 6, 9, 12, 15d	DAI, HE staining, TNF-α, IFN-γ, IL-1β, IL-6, IL-4, intestinal flora and metabolite diversity, PI3K, Akt, VEGFA
Xu XW 2023	Male BALB/c mice, 6–8 weeks, 20 ± 2 g	3% DSS for 9 days, 9 days a cycle, 1 cycle		5/5/5	A. Normal control B. Model control C.DSS + RSV	100 mg/kg/day	RSV was gavaged daily for 9 days after modeling	0-9d	Weight, DAI, HE staining, gross morphology, PCNA, occludin, claudin 1, IL-10, IL-1β, IL-6, TNF-α, intestinal flora and metabolite diversity
Pan HH 2020	Male C57BL/6 mice, 5 weeks, 17–19 g	3% DSS for 7 days, 14 days a cycle, 2 cycle		12/12/12/12	A. Normal control B. Model control C.DSS + RSV D.DSS + 5-ASA (200 mg/kg/day)	100 mg/kg/day	RSV was gavaged daily for 28 days after modeling	DAI: 6, 11, 16, 21, 28days Weight: 5, 10, 15, 20, 25, 28d	Weight, DAI, HE staining, TNF-α, IL-6, IL-1β, colon length, occludin, ZO-1, LC3B, Beclin1, structure of IECs and autolysosomes
Wang JY 2020	Male BALB/c mice, 6–8 weeks	3% DSS for 10 days, 10 days a cycle, 1 cycle		6/6/6	A.Normal control B.Model control C.DSS + RSV	100 mg/kg/day	RSV was gavaged daily for 10 days after modeling	0-10d	Weight, DAI, colon length, HE staining, SUMO1, PCNA, IL-6, IL-8, IL-10, TNF-α, IL-1β
Gu QP 2019	Healthy SD rats, aged 8 weeks, weighing 203–252 g	5% TNBS with anhydrous ethanol (1:1) instilled into colon		12/12/12/12	A.Normal control B.Model control C.DSS + RSV D.DSS + mesalazine (2 g/kg/day)	100 mg/kg/day	RSV was gavaged daily for 7 days	—	DAI, PGE_2_, NO, IL-1β, TNF-α, SIRT1, AMPK, NF-κB p65
Cao LJ 2023	Male C57BL/6 mice	2% DSS for 7 days, 21 days a cycle, 2.5 cycle		6/6/6	A.Normal control B.Model control C.DSS + RSV	100 mg/kg/day	RSV was gavage daily for 49 days after modeling	1-47days, every other day	DAI, weight, colon length, HE staining, PI3K, p-Akt, Akt p-Akt/Akt ratio
Oral administration via vehicle									
Abdin AA 2013	Wistar albino rats, 170–210 g		1.1 mL 7.5 mg/ml OXZ in 40% aqueous ethanol instilled into colon	10/10/10/10	A.Normal control B.Model control + vehicle of 1% CMC C.OXZ+ native RSV D.OXZ + colon-specific delivery RSV	10 mg/kg/day	Native RSV or colon-specific delivery RSV in 1%CMC were gavaged daily for 14 days	—	DAI, HE staining, SphK1, MPO, caspase-3, correlation between DAI and SphK1, MPO, caspase-3, electrophoretic pattern of DNA fragmentation, correlation between histopathological score and SphK1 activity
Yildiz G 2015	Wistar Albino female rats, 250–300 g		Intrarectal administration of a 25 mg TNBS in 37% ethanol	7/7/7/7/7	A. Normal control B. Model control C. RSV (10 mg/kg/day) D.TNBS + DMSO E.TNBS + RSV	10 mg/kg/day	RSV in 0.2 ml DMSO was intraperitoneally daily for 5 days before the induction of colitis	—	HE staining, Cu, Zn-SOD, MDA, GSH-Px, MPO, CAT
Yao J 2011	Male BALB/c mice, 4 weeks, 18–20 g		5% DSS for 7 days, 14 days a cycle, 1 cycle	10/10/10/10	A. Normal control B. Model control C. DSS + RSV D.DSS + polydatin (60 mg/kg/day)	35 mg/kg/day	RSV in 0.5% ethyl alcohol for 14 days through an oral-gastric tube daily after modeling	1-14d	DAI, HE staining, MPO, NF-κB, NF-κB p65, TNF-α, IL-6, IL-1β
Yao J 2010a	Male BALB/c mice, 18–20 g	5% DSS, 7 days, 14 days a cycle, 1 cycle		10/10/10/10	A. Normal control B. Model control C. DSS+ Lose-dose RSV D. DSS+ High-dose RSV	30 mg/kg/day and 60 mg/kg/day	RSV in 0.5% ethyl alcohol, was gavaged daily for 14 days after modeling	1-14d	DAI, HE staining, TNF-α, IFN-γ, IL-8, gp91^pho^, p22^phox^, MDA, MPO, SOD, GSH-Px
Yao J 2010c	Male BALB/c mice, 6–8 weeks	5% DSS for 7 days, 14 days a cycle, 1 cycle		10/10/10/10	A.Normal control B.Model control C.DSS + Low-dose RSV D.DSS + High-dose RSV	40 mg/kg/day and 80 mg/kg/day	RSV in 0.5% ethyl alcohol were gavaged daily for 14 days after modeling	1-14d	DAI, HE staining, IL-10 TNF-α, IL-1β, IL-6
Liu X 2019	Male C57BL/6 mice, 6–8 weeks	1% DSS for 7 days, 14 days a cycle, 1.5 cycle		7/7/7/7	A. Normal control B. Model control C. RSV (80 mg/kg/day) D.DSS + RSV	80 mg/kg/day	RSV in 0.5%CMC-Na were gavaged daily for 7 days (from d 15–21)	0, 1, 2, 3w	Weight, general condition, colon/spleen length/body weight, HE staining, miRNA-31, cyclin D1
Singh UP 2010	Female C57BL/6 mice, 8–12 weeks	3% DSS for 7 days, 14 days a cycle, 1 cycle		6/6/6/6/6/6	A. Normal control B. RSV C. DSS + Low-dose RSV D. DSS + Moderate-dose RSV E.DSS + High-dose RSV F.DSS + vehicle	10 mg/kg/day, 50 mg/kg/day and 100 mg/kg/day	RSV was gavaged daily for 14 days after modeling	1-14d	Weight loss, HE staining, SAA, IL-6, IL-1β, IFN-γ, TNF-α, inflammation scores, colon length, CD4^+^ T cells with IFN-γ/TNF-α, macrophages/T cells percentage, COX1/2, SIRT1, p-IκBα
Yao J 2015	Male BALB/c mice, 6–7 weeks	5% DSS for 7 days, 14 days a cycle, 1 cycle		10/10/10/10	A. Normal control B. Model control C.DSS + Lose-dose RSV D.DSS + High-dose RSV	50 mg/kg/day and 100 mg/kg/day	RSV in 0.5% ethyl alcohol was gavaged daily for 14 days from the 7th d	7-14d	DAI, HE staining, CD4^+^ IL-17^+^ (Th17)/CD4^+^ lymphocytes, CD4^+^ Foxp3^+^ (Treg)/CD4^+^ lymphocytes, IL-6, IL-17, IL-10, TGF-β1, HIF-1α, mTOR, STAT3
Yao J 2010b	Male BALB/c mice, 6–8 weeks	5% DSS for 7 days, 14 days a cycle, 1 cycle		10/10/10/10	A.Normal control B.Model control C.DSS + Low-dose RSV D.DSS + High-dose RSV	50 mg/kg/day and 100 mg/kg/day	RSV in 0.5% ethyl alcohol was gavaged daily for 7 days (from d 8–14)	—	HE staining, CD4^+^CD25^+^ Foxp3+/CD4+ in peripheral blood and mesentery
Ma SG 2019	Male C57/B6 mice, 22–25 g	5% DSS for 7 days, 14 days a cycle, 1 cycle		10/10/10/10	A. Normal control B. Model control C.DSS + Low-dose RSV D.DSS + High-dose RSV	50 mg/kg/day and 100 mg/kg/day	RSV in 0.5% ethyl alcohol was gavaged daily for 7 days (from d 8–14)	—	HE staining, SOD, GSH, ROS, MDA, SIRT3
Zhou XJ 2023	Male BALB/c mice, 6–8 weeks	3% DSS for 7 days, 7 days a cycle, 1 cycle		20/20/20	A. Normal control B. Model control C.DSS + RSV	100 mg/kg/day	RSV in 10% ethanol was gavaged daily for 7 days after modeling	0-7d	DAI, weight, colon length, HE staining, spleen appearance, IL-1β, IL-6, TNF-α, IL-10, ANRIL, Mir-34a, MUC2, GALNT7
Alrafas HR 2020	Female BALB/c mice aged 8–10 weeks	Injection of 1 mg TNBS in 0.1 ml of 50% ethanol		5/5/5/5	A. Normal control B. TNBS + CMC C. TNBS + RSV D. RSV(100 mg/kg/day)	100 mg/kg/day	RSV in 1% CMC was gavaged 24 h prior to TNBS injection for 5 days	0-5d	Weight, colon length, HE staining, representative colonoscopies, T cell distribution, miR profile of cells, FoxP3, IPA, miR-31, Let-7a, miR-132
Alrafas HR 2019	Female BALB/c mice, 6–8 weeks	Intrarectal injections of 1 mg TNBS in 0.1 ml of 50% ethanol		5/5/5/5	A.Normal control B.TNBS + CMC C.TNBS + RSV D.RSV(100 mg/kg/day)	100 mg/kg/day	RSV in 1% CMC cellulose was gavaged daily 24 h prior to TNBS injection for 5 days	0-5d	Weight, survival, colon length, SAA, Lcn2, MPO, colonoscopic examination, HE staining, PAS staining, T cell subsets, CD3, CD4^+^ and CD8^+^ T cells, CD4^+^FOXP3^+^ and CD4^+^IL10^+^ cells population, CD4^+^IFN𝛾^+^ and CD4^+^IL17^+^ cells, intestinal flora and metabolite diversity, SCFAs
Gu QP 2021	C57BL/6 mice, male/female each, 8 weeks,20–25 g	5% DSS for 7 days, 14 days a cycle, 1 cycle		10/10/10/10/10	A. Normal control B. Model control C. DSS + Low-dose RSV D. DSS + High-dose RSV E.DSS + SASP (16 mg/ml)	80 mg/kg/day and 160 mg/kg/day	RSV in 0.5% CMC Na were gavaged daily for 14 days	—	Proportion of Treg/Th17 in T lymphocytes, Treg/Th17 ratio, IL-17/IL-23 in intestinal mucosal tissue, IL-1β, IL-6, TNF-α
Oral administration in standard dietary form									
Larrosa M 2009	Male Fischer F344 rats, 175–200 g	5% DSS for last 5 days of the experiment (from 20 to 25 days), 5 days a cycle, 1 cycle		8/8/8	A. Normal control B. Model control C. DSS + RSV	1 mg/kg/day	Standard chow supplemented with RSV for 25 days after modeling	25d	Weight gain, food/water intake, hematological and serobiochemical parameters, colon length, HE staining, COX2, PTGES, NO, PGE_2_, TBARS, FRAP, intestinal flora diversity
Li F 2021	Male CD-1 mice, 6 weeks	1.5% DSS for 4 days, 11 days a cycle, 4 cycle		10/10/10/10	A. Normal control B. Model control C. PTE (2.3 mg/kg/day) D. DSS + PTE	21 mg/kg/day	0.025% PTE supplemented standard AIN- 93G diet for 44 days after modeling	7, 11, 18, 22, 29, 33, 40, 44d	DAI, HE staining, weight gain, final weight, colon length, IFN-γ, IL-2, IL-4, IL-6, IL-10, intestinal flora and metabolite diversity
Li F 2020	Male CD-1 mice, 6–8 weeks	1.5% DSS for 4 days, 11 days a cycle, 4 cycle		10/10/10/10	A. Normal control B. Model control C.RSV (2.3 mg/kg/day) D.DSS + RSV	21 mg/kg/day	0.025% RSV supplemented standard AIN- 93G diet for more than 40 days after modeling	7, 11, 18, 22, 29, 33, 40, 44d	DAI, HE staining, weight gain, final weight, colon weight/length ratio, GM-CSF, IFN-γ, IL-10, IL-2, IL-1β, IL-6, KC/GRO, TNF-α, intestinal flora and metabolite diversity
Cui XL 2010	Male and female C57BL/6 mice, 8–12 weeks, 20–25 g	1% DSS for 7 days, 14 days a cycle, 2.5 cycle		150ppm: 5/5/5/5 300ppm: 5/10/10	A. Normal control B. Model control C.DSS + Low-dose RSV D.DSS+ High-dose RSV E.RSV	150ppm (21 mg/kg/day) and 300ppm (42 mg/kg/d)	AIN-93G based diet containing RSV for 63 days (from d 8–70)	/	HE staining, colon length, numbers of CD3^+^ T cells express TNF-α and IFN-γ, percentage changes in mucosal neutrophil expression, iNOS, COX-2, TNF-α, p53
Mayangsari Y 2018	Male BALB/c mice, 7 weeks	2% DSS for 8 days, 8 days a cycle, 1 cycle		7/7/7	A. Normal control B. Model control C. DSS + RSV	120 mg/kg	AIN-93G based diet containing 0.1% RSV for 14 days before and during DSS treatment	0–8d	Weight, DAI, HE staining, colon length, LBP, ZO-2, occludin, JAM-A, claudin-2/3/4/7, IL-6, CXCL-2, IL-1β, MCP-1, TNF-α, IL-17A, Ly6G-positive cells

DSS, dextran sulfate sodium; RSV, resveratrol; DAI, Disease Activity Index; HE staining, Hematoxylin‐Eosin staining; TNF‐;α, tumour necrosis factor alpha; IFN‐γ, interferon‐gamma; IL‐1β, interleukin‐1beta; IL‐2, interleukin‐2; IL‐6, interleukin‐6; IL‐4, interleukin‐4; IL‐8, interleukin‐8; IL‐10, interleukin‐10; IL‐17, interleukin‐17; gp91pho, nicotinamide adenine dinucleotide phosphate oxidase membrane subunit gp91pho; p22phox, nicotinamide adenine dinucleotide phosphate oxidase membrane subunit p22phox; PI3K, phosphoinositide 3‐kinase; Akt, protein kinase B; VEGFA, vascular endothelial growth factor A; MDA, malondialdehyde; MPO, myeloperoxidase; SOD, superoxide dismutase; GSH‐Px, glutathione peroxidase; PTE, trans‐3,5‐dimethoxy‐4‐hydroxystilbene; GM‐CSF, Human granulocyte‐macrophage colony stimulating factor; KC/GRO, murine recombinant growth regulatory oncogenes; COX‐2, cyclooxygenase‐2; PTGES, prostaglandin E synthase; NO, nitric oxide; PGE2, prostaglandin E2; TBARS, thiobarbituric acid reactive substance; FRAP, ferric ion reducing antioxidant power; TGF‐β1, T transforming Growth Factor‐β1; HIF‐1α, hypoxia‐inducible factor‐1α; mTOR, mammalian Target of Rapamycin; STAT3, signal transducer and activator of transcription 3; Th17, T helper cell 17; Foxp3, Forkhead Box P3; NF‐κB, nuclear factor‐k‐gene binding; qRT‐PCR, quantitative reverse transcriptase polymerase chain reaction; SAA, serum amyloid A; COX‐1, cyclooxygenase‐1; SIRT1, NAD‐dependent deacetylase sirtuin‐1; p‐IκBα, phosphorylation nuclear factor κB inhibitory factor α; MLN, mesenteric lymph nodes; LP, intestinal lamina propria lymphocytes; PCNA, proliferating cell nuclear antigen; ZO‐1, zonula occludens‐1; LC3B, microtubule associated protein 1 light chain 3 beta; Beclin 1, autophagy gene beclin 1; IECs, intestinal epithelium cell; LBP, lipopolysaccharide binding protein; ZO‐2, zonula occludens‐2; JAM‐A, a type of intercellular tight junction protein; CXCL‐2, chemokine (C‐X‐C motif) ligand 2; MCP‐1, monocyte chemotactic protein 1; SUMO1, small ubiquitin‐like modifier‐1; IPA, ingenuity pathway analysis; ANRIL, antisense non‐coding RNA in the INK4 locus; MUC2, recombinant mucin 2; GALNT7, UDP‐N‐acetyl‐alpha‐D‐galactosamine:polypeptide N‐acetylgalactosaminyltransferase 7; SCFAs, short‐chain fatty acids; CAT, chloramphenicol acetyltransferase; iNOS, inducible nitric oxide synthase; p53, tumor protein 53; PGD2, Prostaglandin D2; SphK1, colonic sphingosine kinase 1; GAPDH, recombinant glyceraldehyde‐3‐phosphate dehydrogenase; TLR4, toll‐like receptor 4 polypeptide; MYD88, myeloid differentiation factor 88; ROS, reactive oxygen species; Atg12, autophagy related 12 homolog; LC3, rabbit anti‐LC3; AMPK, Adenosine 5′‐monophosphate (AMP)‐activated protein kinase.

In terms of the administration mode of resveratrol, 10 studies utilized resveratrol dissolved in water, 5 studies adopted resveratrol combined with a standard diet,. Another 13 studies administered resveratrol via vehicle routes.

### 3.3 Risk of bias assessment

The results of the literature quality assessment are presented in Table 2. Overall, the quality of the included studies was acceptable, with more than half of the studies demonstrating low risk in five or more items. All the studies underwent peer review and showed a low risk in terms of the intrinsic neuroprotective properties of anesthetics. Specifically, temperature control was considered in 14 studies during animal breeding, the randomization of animals in the methodological design was clarified in 20 studies, blinding in the evaluation of outcome measures was adopted in 11 studies, adherence to animal welfare regulations was explicitly stated in 19 studies, and no possible conflicts of interest were declared in 14 studies. However, none of the studies reported the use of blinded modeling, representative animal-specific samples, or the calculation of sample size.

**TABLE 2:** Risk of bias summary.

Study	1	2	3	4	5	6	7	8	9	10
Larrosa M 2009	+	+	+	?	?	+	?	?	+	?
Li F 2021	+	+	+	?	?	+	?	?	+	+
Li F 2020	+	+	+	?	?	+	?	?	+	+
Yao J 2010c	+	?	+	?	?	+	?	?	?	?
Yao J 2011	+	+	+	?	+	+	?	?	+	?
Zhu F 2021	+	?	+	?	?	+	?	?	+	?
Xu XW 2023	+	?	+	?	?	+	?	?	+	+
Pan HH 2020	+	+	+	?	+	+	?	?	+	+
Mayangsari Y 2018	+	+	+	?	+	+	?	?	+	+
Wang JY 2020	+	?	+	?	?	+	?	?	+	+
Alrafas HR 2020	+	+	?	?	?	+	?	?	+	+
Zhou XJ 2023	+	?	?	?	?	+	?	?	+	+
Alrafas HR 2019	+	?	?	?	?	+	?	?	+	+
Yildiz G 2015	+	+	?	?	+	+	?	?	+	+
Cui XL 2010	+	?	?	?	+	+	?	?	+	+
Martín AR 2004	+	+	+	?	+	+	?	?	+	?
Abdin AA 2013	+	?	?	?	?	+	?	?	+	?
Wang JH 2021a	+	?	+	?	?	+	?	?	+	+
Wang JH 2021b	+	+	+	?	+	+	?	?	+	+
Yao J 2010b	+	?	+	?	+	+	?	?	?	?
Liu X 2019	+	?	+	?	?	+	?	?	?	?
Gu QP 2021	+	+	+	?	+	+	?	?	?	?
Cao LJ 2023	+	?	+	?	?	+	?	?	?	?
Ma SG 2019	+	?	+	?	+	+	?	?	?	?
Gu QP 2019	+	+	+	?	?	+	?	?	?	?
Yao J 2010a	+	+	?	?	+	+	?	?	?	?
Yao J 2015	+	+	+	?	?	+	?	?	+	+
Singh UP 2010	+	?	?	?	?	+	?	?	?	?

(Lee and Eun, 2022) peer-reviewed journal; (Ng et al., 2017) temperature control; (Mak et al., 2020) animals were randomly allocated; (Agrawal and Jess, 2022) blind established model; (Aniwan et al., 2022) blinded outcome assessment; (Seyedian et al., 2019) anesthetics used without marked intrinsic neuroprotective properties; (Mattar et al., 2011) animal model (diabetic, advanced age or hypertensive); (Sato et al., 2023) calculation of sample size; (Keller et al., 2019) statement of compliance with animal welfare regulations; (Piechota-Polanczyk and Fichna, 2014) possible conflicts of interest.

### 3.4 The role of resveratrol in the treatment of IBD

#### 3.4.1 Histopathological index

13 out of the 28 preclinical studies included in the analysis reported histopathological index as an outcome measure. Upon extraction and aggregation of these data, the results revealed that the histopathological index at the end of the experiment was effectively controlled and significantly lower in the resveratrol treatment group compared to the model group (*n* = 147/95, WMD = −2.58 [-3.29, −1.87], *p* < 0.00001; Table 3). Relevant forest plot was provided in the Supplementary Figure S1.

**TABLE 3:** Subgroup analysis of histopathological index.

Outcomes			Studies	Sample size (R/C)	WMD (95% CI)	*p*-value
Histopathological index			13	147/95	−2.58 [-3.29, −1.87]	<0.00001
Types of animals						
BALB/c mice			6	92/52	−1.99 [-2.98, −1.00]	<0.0001
C57BL/6 mice			2	18/11	−5.28 [-10.58, 0.02]	0.05
Wistar albino rats			1	10/5	−1.52 [-1.73, −1.30]	<0.00001
Fischer F344 rats			1	8/8	−2.01 [-3.25, −0.77]	0.002
CD-1 mice			2	12/12	−3.31 [-4.15, −2.46]	<0.00001
Sex of animals						
Male			11	132/85	−1.98 [-2.47, −1.49]	<0.00001
Female			1	5/5	−7.21 [-7.73, −6.70]	<0.00001
Th length of the molding cycle						
≤14 days			7	107/60	−1.40 [-1.86, −0.95]	<0.00001
> 14 days			4	25/25	−3.97 [-5.45, −2.48]	<0.00001
Modeling method						
OXZ			1	10/5	−1.52 [-1.73, −1.30]	<0.00001
TNBS			1	5/5	−7.21 [-7.73, −6.70]	<0.00001
DSS < 3%			5	32/32	−3.46 [-5.05, −1.87]	<0.0001
DSS≥3%			6	100/53	−1.51 [-2.09, −0.93]	<0.00001
Dosage of resveratrol						
≤40 mg/kg			5	40/30	−2.06 [-2.92, −1.20]	<0.00001
40–80 mg/kg			3	37/22	−1.40 [-2.28, −0.52]	0.002
> 80 mg/kg			7	70/43	−3.47 [-4.97, −1.98]	<0.00001
Administration method of resveratrol						
Oral administration of resveratrol dissolved in normal saline			3	30/21	−4.48 [-7.68, −1.28]	0.006
Oral administration of resveratrol dissolved in ethyl alcohol			4	65/35	−2.05 [-3.34, −0.76]	0.002
Oral administration of resveratrol dissolved in CMC			2	17/12	−6.06 [-15.35, 3.22]	0.20
Standard chow supplemented with resveratrol			4	27/27	−2.41 [-3.79, −1.02]	0.0006

CI, confidence interval; R/C, resveratrol/control; WMD, weighted mean difference; OXZ, oxazolone; TNBS, 2,4,6-trinitrobenzenesulfonic acid; DSS, dextran sulfate sodium salt; CMC, carboxy methylcellulose.

#### 3.4.2 Subgroup analysis of histopathological index

A subgroup analysis was conducted on histopathological index, with six grouping criteria considered and all 13 studies that reported histopathological index included in the analysis. The findings revealed that high doses (>80 mg/kg) of resveratrol (7 studies, n = 70/43, WMD = −3.47 [-4.97, −1.98], *p* < 0.00001; Table 3) had the most significant control effect on histopathological index. Interestingly, incorporating resveratrol into the standard diet (4 studies, n = 27/27, WMD = −2.41 [-3.79, −1.02], *p* < 0.0006; Table 3) showed a low therapeutic effect similar to that of dissolved in alcohol (4 studies, n = 65/35, WMD = −2.05 [-3.34, −0.76], *p* < 0.002; Table 3) and significantly less than that of dissolved in water (3 studies, n = 30/21, WMD = -4.48 [-7.68, −1.28], *p* < 0.006) or in CMC (2 studies, n = 27/27, WMD = −2.41 [-3.79, −1.02], *p* < 0.0006; Table 3). However, due to the small number of studies included in each administration mode, it is difficult to make a judgment. Consequently, no definitive conclusions were reached regarding the impact of animal sex, animal species, or modeling method.

Notably, subgroup analyses of molding cycle revealed a more significant decline in histopathological index within the long-cycle group (4 studies, n = 25/25, WMD = −3.97 [-5.45, −2.48], *p* < 0.00001; Table 3). In terms of modeling methods, the subgroup using low-concentration DSS (6 studies, n = 100/53, WMD = −1.51 [-2.09, −0.93], *p* < 0.00001; Table 3) showed lower histopathological index compared to the high-concentration DSS group. Relevant forest plots were provided in the Supplementary Figure S2.

#### 3.4.3 Final DAI score, final weight change and other histopathological indicators

The final DAI score was reported in 17 preclinical studies. The aggregate results indicated a significantly lower DAI score in the resveratrol group compared to the model group, suggesting that resveratrol effectively alleviates inflammation associated with IBD (*n* = 278/155, WMD = −1.75 [-2.09, −1.41], *p* < 0.00001; Table 4). 6 studies examined the final weight change, and our analysis showed resveratrol was effective in maintaining weight in IBD animals (*n* = 108/49, WMD = 10.33 [9.96, 10.70], *p* < 0.001; Table 4). In terms of pathological indicators other than histopathological index, we summarized SI data from 4 studies, and the results showed that resveratrol can reduce SI (*n* = 43/33, WMD = −0.52 [-0.67, −0.37], *p* < 0.00001; Table 4). Besides, 6 studies measured colon length, and analysis of the data manifested that the colon length of the resveratrol group was found to be significantly longer than that of the model group (*n* = 116/79, WMD = 1.17 [0.76, 1.57], *p* < 0.00001; Table 4). Relevant forest plots were provided in the Supplementary Figure S3.

**TABLE 4:** Final DAI score, final weight change and other histopathological indicators.

Outcomes	Study	Sample size (R/C)	WMD (95% CI)	*p*-value
Final DAI score	17	278/155	−1.75 [-2.09, −1.41]	<0.00001
Final weight change (%)	6	108/49	10.33 [9.96, 10.70]	<0.00001
Colon length	9	116/79	1.17 [0.76, 1.57]	<0.00001
SI	4	43/33	−0.52 [-0.67, −0.37]	<0.00001

DAI, disease activity index; R/C, resveratrol/control; WMD, weighted mean difference; CI, confidence interval; SI, the spleen index.

#### 3.4.4 Effects of resveratrol on inflammatory indicators

16 studies reported TNF-α, and combined data showed that resveratrol significantly reduced levels of this inflammatory factor in IBD animals (*n* = 251/153, SMD = −2.85 [-3.69, −2.02], *p* < 0.00001; Table 5). 15 studies selected IL-6 as an outcome indicator, and the summary results demonstrated a significant reduction in the resveratrol group compared to the model group (n = 237/175, SMD = −5.15 [-6.40, −3.90], *p* < 0.00001; Table 5). Similarly, 15 studies examined IL-1β, with results consistent with the previously mentioned factors (n = 238/126, SMD = −3.38 [-4.40, −2.36], *p* < 0.00001; Table 5). 3 studies investigated IL-8, and we found consistent results upon pooling the data (n = 46/26, SMD = −2.85 [-4.92, −0.78], *p* < 0.00001; Table 5). Additionally, 4 studies analyzed IFN-γ, and the result showed a decrease in this inflammatory factor (n = 42/32, SMD = −4.04 [-6.50, −1.58], *p* = 0.001; Table 5).

**TABLE 5:** Inflammatory indicators, oxidative stress-related index and enzyme metabolites.

Outcomes	Study	Sample size (R/C)	SMD (95% CI)	*p*-value
Inflammatory indicators				
TNF-α	16	251/153	−2.85 [-3.69, −2.02]	<0.00001
IL-8	3	46/26	−2.85 [-4.92, −0.78]	= 0.007
IL-6	15	237/135	−5.15 [-6.40, −3.90]	<0.00001
IL-10	10	182/102	3.51 [2.12, 4.90]	<0.00001
IFN-γ	4	42/32	−4.04 [-6.50, −1.58]	= 0.001
IL-1β	15	238/126	−3.38 [-4.40, −2.36]	<0.00001
Oxidative stress-related index				
MDA	3	47/27	−1.43 [-2.15, −0.71]	= 0.0001
MPO	6	71/46	−1.62 [-2.09, −1.15]	<0.00001
SOD	3	47/27	24.99 [14.40, 35.57]	<0.00001
Enzyme metabolites				
PGE_2_	3	44/28	−236.85 [-323.88, −149.82]	<0.00001

R/C, resveratrol/control; SMD, standard mean difference; WMD, weighted mean difference; CI, confidence interval; TNF-α, tumor necrosis factor-α; IL-8, interleukin-8; IL-6, interleukin-6; IL-10, interleukin-10; IFN-γ, interferon-γ; IL-1β, interleukin-1β; MDA, malondialdehyde; MPO, myeloperoxidase; SOD, superoxide dismutase; PGE_2_, prostaglandin E_2_.

However, IL-10 was assessed in 10 studies, and the sammary of datas indicated that resveratrol increased level of this inflammatory factor in the treatment group (n = 182/102, SMD = 3.51 [2.12, 4.90], *p* < 0.00001; Table 5). Relevant forest plot was provided in the Supplementary Figure S4.

#### 3.4.5 Effects of resveratrol on oxidative stress-related indicators and enzyme metabolites

3 studies reported PGE2, and the aggregate results showed that resveratrol had a downregulation effect on this index (n = 44/28, WMD = −236.85 [-323.88, −149.82], *p* < 0.00001; Table 5). 3 studies that evaluated MDA and summarized the extracted data also showed a decline (n = 47/27, SMD = −1.43 [-2.15, −0.71], *p* = 0.0001; Table 5). 6 studies used MPO as an outcome, and the results were the same when the data were combined (n = 71/46, SMD = −1.62 [-2.09, −1.15], *p* < 0.00001; Table 5).

Furthermore, in terms of the reduction of oxidative stress-related enzyme indexes mentioned above, summary statistics from the 3 studies measuring SOD indicated an upregulation effect (n = 47/27, SMD = 24.99 [14.40, 35.57], *p* < 0.00001; Table 5). Relevant forest plots were provided in the Supplementary Figure S5.

#### 3.4.6 Publication bias

**FIGURE 2:**
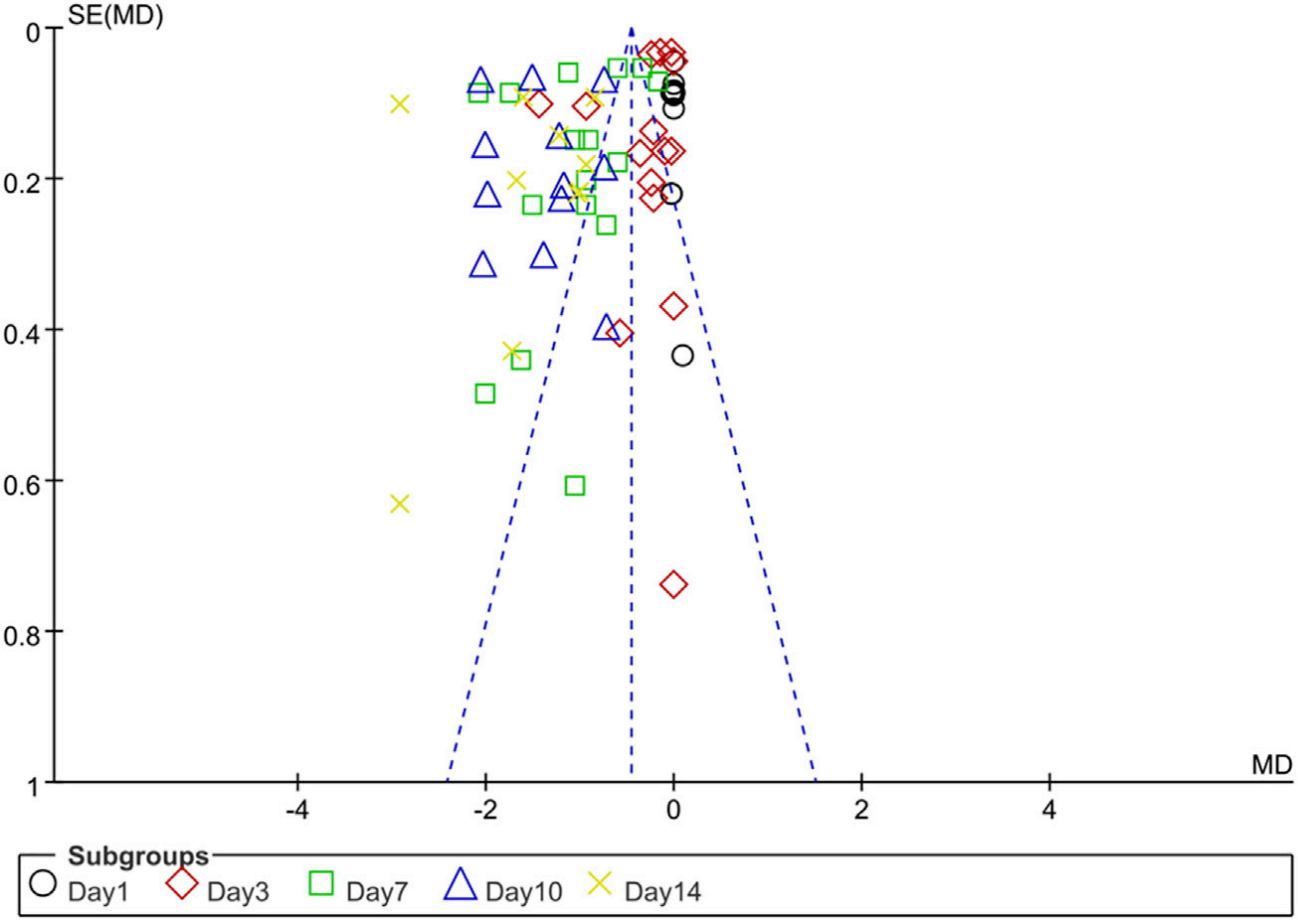
Funnel plots of publication bias for disease activity index.

The funnel plot of publication bias for DAI scores over time after IBD showed significant asymmetry, suggesting that the included studies had a higher likelihood of publication bias (Figure 2).

## 4 Discussion

### 4.1 Summary of main results

In this study, we performed a quantitative meta-analysis to assess the effectiveness of resveratrol in animal models for Inflammatory Bowel Disease (IBD). Twenty-eight studies were included in the study, most of which demonstrated satisfactory results in risk of bias assessment. Analysis of the included preclinical studies revealed that resveratrol significantly decreased the severity of IBD compared to controls, particularly evident in controlling histopathological index. Subgroup analysis indicated that high-dose resveratrol (>80 mg/kg) had superior efficacy compared to medium-dose resveratrol (>40 mg/kg, ≤80 mg/kg) and low-dose resveratrol (≤40 mg/kg). Furthermore, resveratrol demonstrated effectiveness in alleviating weight loss in IBD animals and improving other pathological indicators like spleen index and colon length. Quantitative analysis of inflammatory factors and oxidative stress highlighted a significant correlation between the therapeutic benefits of resveratrol in IBD and its ability to reduce inflammation and oxidative stress.

### 4.2 Possible mechanism of resveratrol in inflammatory factors

The IBD process triggers the release of inflammatory factors via various signaling pathways among which are the Toll-like receptor pathway, MyD88, mitogen-activated protein kinase (MAPK) pathway, and NF-κB pathway (Xiong et al., 2022; Zhu et al., 2023). Additionally, the phosphatidylinositol 3-kinase (PI3K)/protein kinase B (AKT) pathway also contributes to this process (Shen et al., 2020; Zhang Z. et al., 2022).

TLR4 can stimulate the release of inflammatory mediators and activate the innate immune response (Feng et al., 2016). MyD88 is pivotal in relaying upstream signals (Zhou et al., 2018). The modeling reagent triggers TLR4 activation, which results in a marked increase in the phosphorylation of ERK (a member of the MAPK family), IκBα (an inhibitor of NF-κB), and mammalian target of rapamycin (mTOR) via MyD88-dependent signaling (Shen et al., 2020). Subsequently, the phosphorylation of IκBα promotes its degradation, thereby activating the transcription of genes involved in the NF-κB inflammatory cascade (Wang et al., 2022). PI3K engages and doubly phosphorylates the downstream signaling protein Akt through specific key metabolites (Vanhaesebroeck et al., 2010). mTOR, a downstream effector of Akt, modulates the pro-inflammatory activity of the NF-κB pathway and suppresses autophagy (Thorpe et al., 2015; Xianjuan et al., 2021). Moreover, activated Akt directly enhances the phosphorylation of NF-κB, leading to the transcription of inflammatory factors, such as TNF-α, which disturbs the cytokine balance and intensifies inflammatory responses (Arranz et al., 2012; Vergadi et al., 2017). The activation of TNF-α and IL-6 in the intestinal mucosa initiates a positive feedback loop in the NF-κB pathway. IL-6 facilitates this cycle by promoting the phosphorylation of signal transducer and activator of transcription 3 (STAT3) (Grivennikov et al., 2009).

We quantitatively analyzed the data related to inflammatory factors, and TNF-α, IL-6, IL-1β, IL-8, IFN-γ and IL-10 all showed positive results. Our findings suggest that resveratrol may exhibit anti-inflammatory effects in Inflammatory Bowel Disease (IBD) by increasing IL-10 levels and decreasing TNF-α, IL-6, IL-1β, and IL-8 levels.

### 4.3 Possible mechanism of resveratrol for alleviating oxidative stress response

The sirtuin (SIRT) family plays a protective role in cells by defending against oxidative stress damage. Specifically, SIRT3 boosts the activity of reactive oxygen species (ROS) scavenging enzymes, preserves mitochondrial functionality, and prevents ROS buildup within mitochondria, thereby exerting a potent antioxidant effect (Jianheng, 2021). Moreover, In the gastrointestinal tract, a diverse microbial community—encompassing both beneficial and detrimental bacteria—plays a critical role in modulating oxidative stress, which is a significant factor in the pathogenesis of IBD. Probiotic strains such as *Lactobacillus* and Bifidobacterium secrete cytoplasmic superoxide dismutase A (SodA), which mitigates oxidative stress and triggers the upregulation of nuclear factor erythroid 2-related factor 2 (Nrf2)-regulated antioxidant enzymes, thereby decelerating the inflammatory process in the colon (Larrosa et al., 2009; Yao et al., 2011; Hu et al., 2019). However, *Enterococcus faecalis*, found near the surface of the oxygenated colon, produces extracellular O2- at a higher rate, contributing to intestinal tissue damage (Larrosa et al., 2009; Yao et al., 2011; Hu et al., 2019).

**FIGURE 3:**
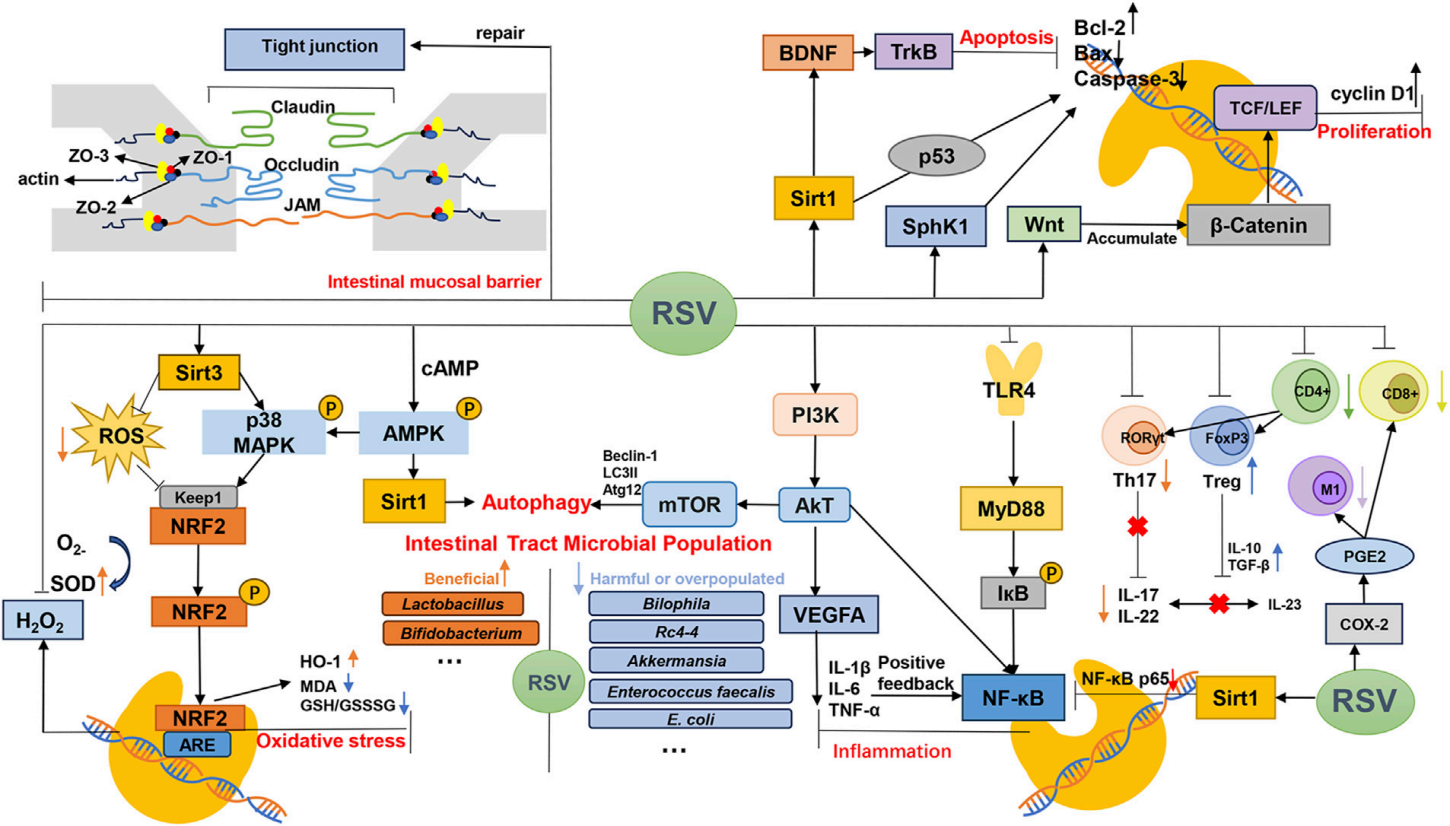
Mechanism of resveratrol for IBD.

Our meta-analyses of MDA, MPO, ROS and SOD were all positive, suggesting that resveratrol may play an antioxidant stress role in IBD. The mechanisms of resveratrol in the treatment of IBD, as outlined in the included literature, have been comprehensively summarized (Table 6). Additionally, corresponding mechanism diagrams have been developed based on these mechanisms (Figure 3).

**TABLE 6:** Mechanism of resveratrol for IBD in the included studies.

Study	Mechanism	Effect
Zhu F 2021	Inflammation, intestinal flora diversity	Decreased TNF-α, IL-1β, IL-6, and IL-8 inhibited protein expressions of inflammatory (PI3K/Akt/VEGFA), regulated *Akkermansia* to steady-state level
Yao J 2010a	Inflammation, oxidative stress	Inhibited inflammatory (TNF-α, IFN-γ, IL-8, gp91^pho^, and p22^phox^), decreased MDA and MPO, increased SOD and GSH-Px
Li F 2021	Inflammation, intestinal flora diversity	Decreased IFN-γ, IL-2, IL-6, increased IL-4, diminished abundance of bacteria (*Bilophila* and *Rc4-4*) that promotes intestinal mucosal damage
Li F 2020	Inflammation, intestinal flora diversity	Decreased GM-CSF, KC/GRO, IFN-γ, IL-2, IL-1β, TNF-α, and IL-6, regulated the structure of gut microbiota (*Akkermansia*, *Bifidobacterium*, *Bilophila*, *Dorea*, and *Sutterella*)
Larrosa M 2009	Inflammation, nitrosative stress, and intestinal flora diversity	Decreased COX-2, NO, and PTGES, increased PGE2, improve gut microbiota composition (*Lactobacillus*, *Bifidobacterium*, *E. coli*)
Yao J 2015	Inflammation	Regulated T cell subset (Th17 lymphocytes, and Treg lymphocytes), decreased IL-6, and IL-17, increased IL-10, and TGF-β1, downregulated HIF-1α, mTOR, and STAT3
Yao J 2010b	Inflammation, oxidative stress	Alleviated oxidative stress (MPO, and GAPDH), inhibited inflammatory (NF-κB, NF-κB p65, TNF-α, IL-6, and IL-1β)
Singh UP 2010	Inflammation	Decreased IL-6, IL-1β, IFN-γ, SAA, p-IκBα, and TNF-α, downregulated number of CD4^+^ T cells expressing IFN-γ or TNF- α, suppressed COX-2 expression, increased SIRT1 expression
Xu XW 2023	Inflammation, intestinal mucosal barrier, intestinal flora diversity	Increased the tight junction proteins Occludin and Claudin 1, decreased IL-10, IL-1β, IL-6, TNF-α, regulated the structure of gut microbiota (*Bacteroidetes*, *Firmicutes*, *Proteobacteria*)
Pan HH 2020	Inflammation, intestinal mucosal barrier, autophagy	Decreased TNF-α, IL-6, IL-1β, increased the expression of occludin and ZO-1, promoted autophagy (LC3B and Beclin1)
Mayangsari Y 2018	Inflammation, intestinal mucosal barrier	Repaired mucosal barrier (LBP, JAM-A, zonula occludens, occluding, and claudins), inhibited inflammatory (IL-6, CXCL-2, IL-1β, MCP-1, TNF-α, and IL-17A), suppressed neutrophil infiltration (Ly6G-positive cells)
Wang JY 2020	Inflammation	Decreased IL-6, IL-8, TNF-α, IL-1β, increased IL-10, inhibited the expression of SUMO1
Alrafas HR 2020	Inflammation	Regulated T cell distribution (CD3, CD4^+^ and CD8^+^ T cells, CD4^+^FOXP3^+^, CD4^+^IL10+and CD4^+^IL17^+^ cells), downregulated miRs (miR-31, Let-7a, and miR-132) that target anti-inflammatory T cell-associated factors
Zhou XJ 2023	Inflammation, intestinal mucosal barrier	Decreased IL-1β, IL-6, TNF-α, increased IL-10, upregulated miR-34a to restore levels of mucin MUC2 and its synthase GALNT7
Yao J 2010c	Inflammation	Increased CD4^+^CD25^+^ Foxp3+/CD4+ in peripheral blood and mesentery
Alrafas HR 2019	Inflammation, intestinal flora diversity	Decreased inflammatory biomarkers (SAA, Lcn2, and MPO), regulated the T cell subsets (CD3^+^, CD4^+^ and CD8^+^ T cells, CD4^+^FOXP3^+^ and CD4^+^IL10^+^ cells, CD4^+^IFNY^+^ and CD4^+^IL17^+^ cells), optimize microbial community structure (*A. muciniphila*, *R. gnavus*, and *B. acidifaciens*)
Yildiz G 2015	Oxidative stress	Increased SOD, GSH-Px, and CAT, decreased MPO, and MDA
Cui XL 2010	Inflammation, nitrosative stress	Decreased the numbers of CD3^+^ T cells that express TNF-α and IFN-γ, downregulated mucosal neutrophil expression, iNOS, TNF-α, and p53, increased COX-2
Martín AR 2004	Inflammation, oxidative stress	Decreased IL-1β, PGD2, and MPO, increased COX-2
Abdin AA 2013	Inflammation, apoptosis	Inhibition of increased SphK1 activity associated with MPO to reduce inflammatory cell infiltration and downregulated the activity of apoptosis-related caspase-3
Yao J 2010days	Inflammation, oxidative stress	Decreased TNF-α, IL-1β, and IL-6, increased IL-10, and GAPDH
Wang JH 2021a	Inflammation	Decreased TNF-α, IL-1β, and IL-6, increased IL-10, inhibited TLR4/MYD88/NF-κB P65 pathway
Ma SG 2019	Oxidative stress	Increased SOD, GSH, SIRT3, decreased ROS, MDA
Wang JH 2021b	Inflammation, autophagy	Increased IL-10, decreased TNF-α, IL-1β, and IL-6, Promoted the activation of the SIRT1/mTOR signaling pathway and reduced the expression of autophagy related proteins (Atg12, Beclin-1, LC3II), upregulated the proportion of CD4+T in MLN
Liu X 2019	Cell proliferation	Suppress Wnt/β- Catenin signaling pathway and downregulated its target protein cyclin D1
Gu QP 2021	Inflammation	Upregulated Treg/Th17 to reduce the expression of IL-17 and IL-23, decreased IL-1β, IL-6 and TNF-α
Gu QP 2019	Inflammation	Decreasd PGE2,NO,IL-1β, and TNF-α, activated the SIRT1/AMPK signaling pathway to inhibit NF- кB p65 protein
Cao LJ 2023	Inflammation	Inhibited the PI3K/Akt signaling pathway, downregulated p-Akt and Akt

TNF-α, tumour necrosis factor alpha; IFN-γ, interferon-gamma; IL-1β, interleukin-1beta; IL-2, interleukin-2; IL-6, interleukin-6; IL-4, interleukin-4; IL-8, interleukin-8; IL-10, interleukin-10; IL-17, interleukin-17; gp91^pho^, nicotinamide adenine dinucleotide phosphate oxidase membrane subunit gp91^pho^; p22^phox^, nicotinamide adenine dinucleotide phosphate oxidase membrane subunit p22^phox^; PI3K, phosphoinositide 3-kinase; Akt, protein kinase B; VEGFA, vascular endothelial growth factor A; MDA, malondialdehyde; MPO, myeloperoxidase; SOD, superoxide dismutase; GSH-Px, glutathione peroxidase; GM-CSF, Human granulocyte-macrophage colony stimulating factor; KC/GRO, murine recombinant growth regulatory oncogenes; COX-2, cyclooxygenase-2; PTGES, prostaglandin E synthase; NO, nitric oxide; PGE2, prostaglandin E2; TGF-β1, T tansforming Growth Factor–β1; HIF-1α, hypoxia-inducible factor-1α; mTOR, mammalian Target of Rapamycin; STAT3, signal transducer and activator of transcription 3; Th17, T helper cell 17; Foxp3, Forkhead Box P3; NF-κB, nuclear factor-k-gene binding; SAA, serum amyloid A; COX-1, cyclooxygenase-1; SIRT1, NAD-dependent deacetylase sirtuin-1; p-IκBα, phosphorylation nuclear factor κB inhibitory factor α; MLN, mesenteric lymph nodes; ZO-1, zonula occludens-1; LC3B, microtubule associated protein 1 light chain 3 beta; Beclin 1, autophagy gene beclin 1; ZO-2, zonula occludens-2; JAM-A, a type of intercellular tight junction protein; CXCL-2, chemokine (C-X-C motif) ligand 2; MCP-1, monocyte chemotactic protein 1; SUMO1, small ubiquitin-like modifier-1; MUC2, recombinant mucin 2; GALNT7, UDP-N-acetyl-alpha-D-galactosamine:polypeptide N-acetylgalactosaminyltransferase 7; Lcn2, Lipocalin 2; CAT, chloramphenicol acetyltransferase; iNOS, inducible nitric oxide synthase; p53, tumor protein 53; PGD2, Prostaglandin D2; SphK1, colonic sphingosine kinase 1; GAPDH, recombinant glyceraldehyde-3-phosphate dehydrogenase; TLR4, toll-like receptor 4 polypeptide; MYD88, myeloid differentiation factor 88; ROS, reactive oxygen species; Atg12, autophagy related 12 homolog; Wnt, AMPK, Adenosine 5‘-monophosphate (AMP)-activated protein kinase.

### 4.4 Dose and formulation of resveratrol for IBD

Subgroup analysis of effective resveratrol dosages for IBD treatment indicates that doses exceeding 80 mg/kg/day may enhance efficacy, particularly in controlling histopathological index. Furthermore, five of the reviewed studies utilized sulfasalazine as a positive control. High doses of resveratrol (>80 mg/kg/day) demonstrated comparable results to positive controls in terms of DAI control and reduction of inflammatory markers (Qiuping et al., 2019; Pan et al., 2020; Jianheng et al., 2021; Qiuping et al., 2021; Zhu et al., 2021). However, due to limited data on doses beyond 80 mg/kg/day, an optimal dosage range cannot be conclusively determined. While higher dosages may offer enhanced benefits, safety concerns may arise. Although resveratrol is generally considered to have low toxicity and good tolerance, the threshold for resveratrol toxicity remains unclear (Hebbar et al., 2005; Almeida et al., 2009; Williams et al., 2009; Gal et al., 2021; Zhang et al., 2021). Therefore, future research could explore a dose gradient with 80 mg as the middle or lower limit to identify the most effective dose without causing toxicity.

Subgroup analyses revealed that resveratrol dissolved in saline or CMC exhibited significantly greater efficacy compared to when incorporated in a standard diet or dissolved in alcohol. However, due to the limited number of included studies, definitive conclusions could not be made. One plausible explanation for this disparity is that incorporating resveratrol into the diet could potentially alter its distribution and absorption in the intestinal mucosa, consequently diminishing its bioavailability. Moreover, dissolving resveratrol in alcohol might lead to additional harm to the intestinal mucosa. It is noteworthy that despite the inclusion of only two studies, using CMC as a carrier notably enhanced the efficacy of resveratrol. This enhancement could be attributed to CMC’s ability to improve the stability of resveratrol suspension and facilitate its penetration in intestinal mucosal tissues. Therefore, the application of resveratrol using CMC as a carrier for IBD is worth further exploration. Additionally, there have been promising preclinical studies using nano-formulations of resveratrol (Lozano-Pérez et al., 2014; Gandhi et al., 2020; Naserifar et al., 2020; Pujara et al., 2021; Li et al., 2023). Although the number of such studies is currently limited, they show potential for addressing the issue of low bioavailability associated with resveratrol. In summary, resveratrol can be considered a highly effective nutritional additive and complementary drug, and its research as a candidate drug for treatment has important practical significance.

### 4.5 Effects of animal sex, types and modeling methods on the efficacy of resveratrol

In the subgroup analysis of sex, only 1 study utilized a female animal model (Alrafas et al., 2019). The result showed that resveratrol significantly reduced the histopathological index in female IBD animals compared to males. However, the evidence is not convincing and should be considered as reference only. As for the subgroup analysis of the types of experimental animals, C57BL/6 mice had the lowest histopathological index among the 4 types considered, although this subgroup only included 2 studies (Pan et al., 2020; Liujing et al., 2023). With a limited number of studies including subgroup analyses in rat models, it remains challenging to determine if the effectiveness of resveratrol differs between IBD rats and mice. When considering modeling methods, resveratrol demonstrated better control of histopathological index in low-concentration DSS animal models (DSS <3%) compared to high-concentration models (DSS ≥3%). While resveratrol showed promising results in animal models using TNBS as a modeling agent, definitive conclusions are hindered by the limited number of studies and other variables. Furthermore, in subgroup analysis based on the length of the modeling cycle, resveratrol exhibited a stronger protective effect on the pathological damage in IBD animals with longer modeling cycles (>14 days), suggesting a potential better efficacy in the chronic stage of intestinal inflammation compared to the acute stage. However, this conclusion is derived from preclinical studies with a limited sample size, warranting caution in interpretation.

### 4.6 Strengths and limitations of this review

To date, no quantitative studies have been conducted to analyze the therapeutic effects of resveratrol on animal models of IBD. This meta-analysis is a comprehensive and extensive inclusion of relevant studies. The inclusion criteria are not limited to the animal type and modeling method, ensuring more objective and representative results. In this review, histopathological index was chosen as the primary outcome indicator for quantitative analysis of the data in the included literature. This index is believed to provide a more precise and direct quantification of the extent and severity of intestinal mucosal pathological damage in IBD. Pathologists evaluate this index using a blind method, ensuring rigor and professionalism. Furthermore, this meta-analysis includes a subgroup analysis to evaluate the efficacy of various resveratrol doses in the treatment of IBD. Currently, there is a lack of in-depth clinical trials examining the dose-effect relationship of resveratrol in the treatment of IBD. Therefore, this study can provide certain dose evidence of IBD animal models for clinical trials. In subgroup analysis, we aimed to investigate the impact of resveratrol administration routes, IBD modeling methods, and modeling duration on histopathological index. By analyzing inflammatory factors and oxidative stress index, this study summarizes the specific mechanism of resveratrol in treating IBD with a larger sample size of animals.

However, this review has several limitations. Firstly, the small number of included studies may have overlooked unpublished or recently emerged animal studies, precluding the establishment of an upper limit for resveratrol administration based on the current data. Secondly, due to insufficient literature included, subgroup analysis of factors such as the sex of experimental animals, animal types, and modeling methods were limited. Thirdly, the presence of significant publication bias, as indicated by the funnel plot, should not be ignored. Last but not least, most of the animal studies we included used mice model. Although the mice model is the most established model for studying IBD, it does have clear limitations. Using mice model cannot dynamically track the pathological development of IBD. Mice have genetic and immune system differences compared to humans (Wen et al., 2024). Moreover, the longer pregnancy and growth cycle of mice increases the time and cost of experimental modeling (Kim et al., 2012). These limitations somewhat hinder the advancement of IBD research and the clinical translation of IBD treatments.

The current literature suggests that the zebrafish IBD model holds significant promise, particularly due to its high reproductive rate and short lifecycle, which can enhance experimental efficiency and reduce costs (Flores et al., 2020). Additionally, the transgenic strain exhibits fluorescence protein expression, facilitating the use of *in vivo* imaging technology to monitor intestinal tissue pathology in IBD (Marjoram and Bagnat, 2015; Hanyang et al., 2017). Zebrafish share genetic, digestive system, and immune system similarities with humans, making them a potential alternative to the conventional mouse model in future preclinical studies of IBD.

## 5 Conclusion

Our study demonstrated that resveratrol had a significant effect on reducing disease severity in animal models of IBD. This positive effect was observed through various indicators such as inflammation, general condition, histopathology, and oxidative stress. Resveratrol achieves improvements in these indicators by acting as an antioxidant, anti-inflammatory, and immunomodulatory agent. Notably, the effectiveness of high-dose resveratrol in promoting IBD disease remission was more pronounced compared with low-dose resveratrol. As a result, resveratrol shows promise as a potential candidate for future IBD clinical trials. However, it is important to interpret the results of this pre-clinical review with caution due to limitations in animal experimental methods and the quality of evidence.

## Data Availability

The original contributions presented in the study are included in the article/Supplementary Material, further inquiries can be directed to the corresponding authors.
