# Supplementary material for: Resveratrol for inflammatory bowel disease in preclinical studies: a systematic review and meta-analysis
Source: Front Pharmacol. 2024 Jun 14;15:1411566. doi: 10.3389/fphar.2024.1411566 (PMC11211549; doi:10.3389/fphar.2024.1411566)
Supplement: Supplementary file 1 [file DataSheet1.docx]

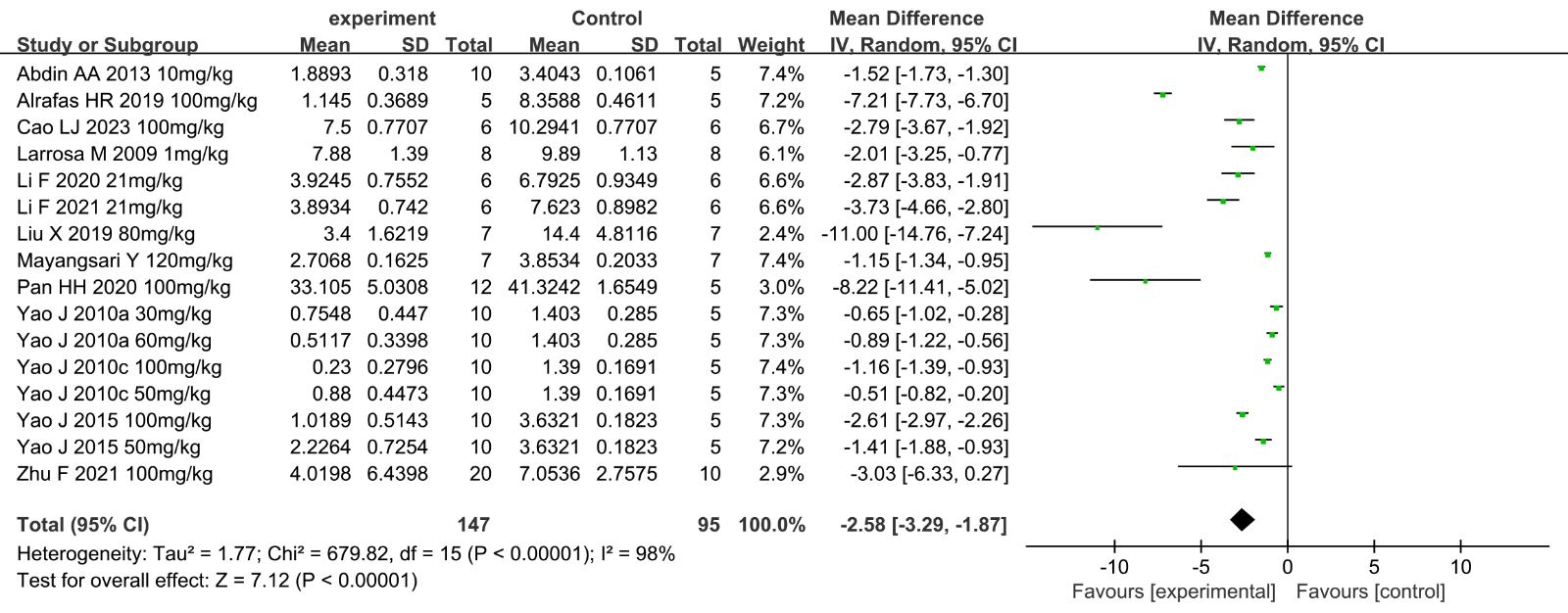


Figure S1. Effect of resveratrol on histopathological index.

A


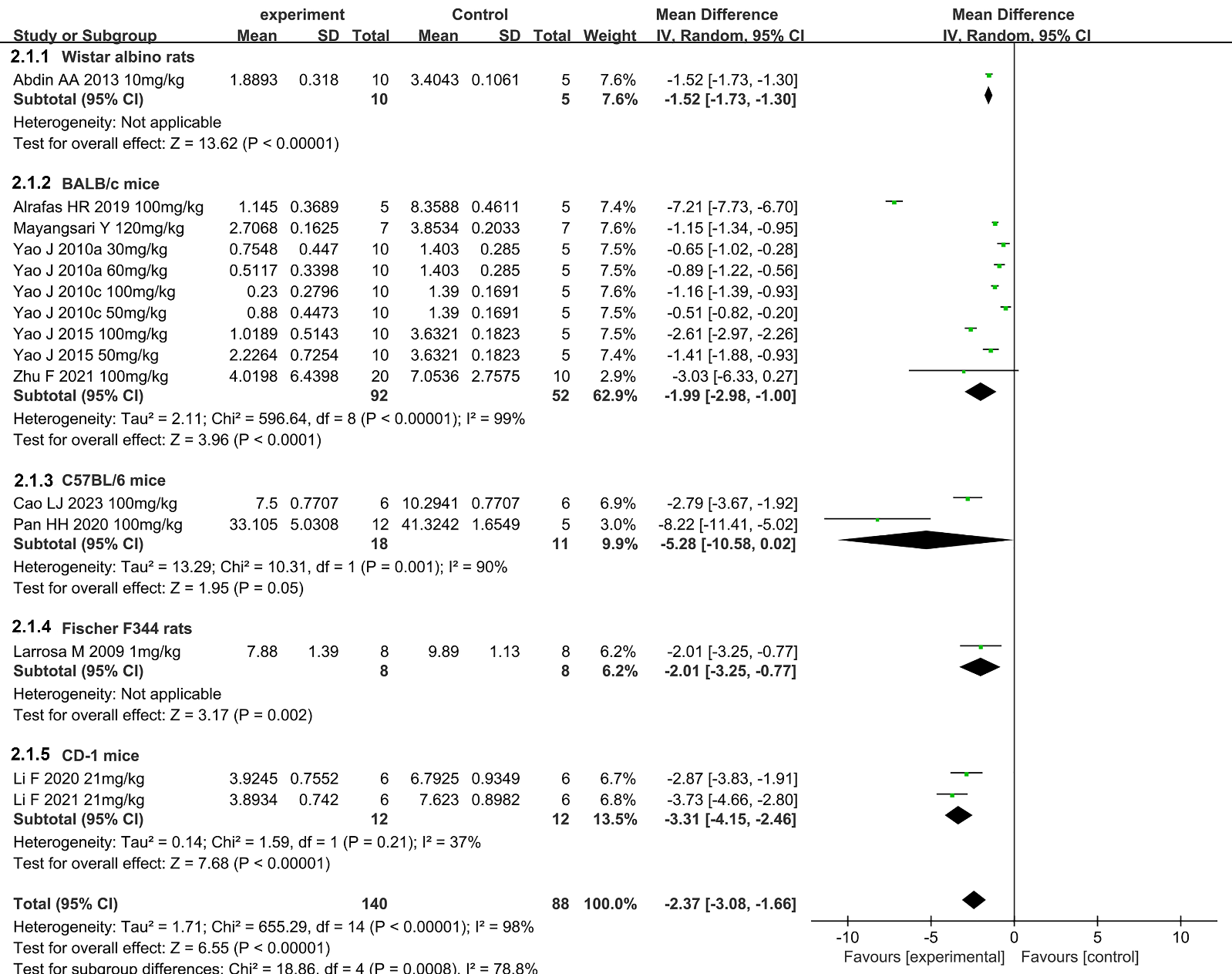


B


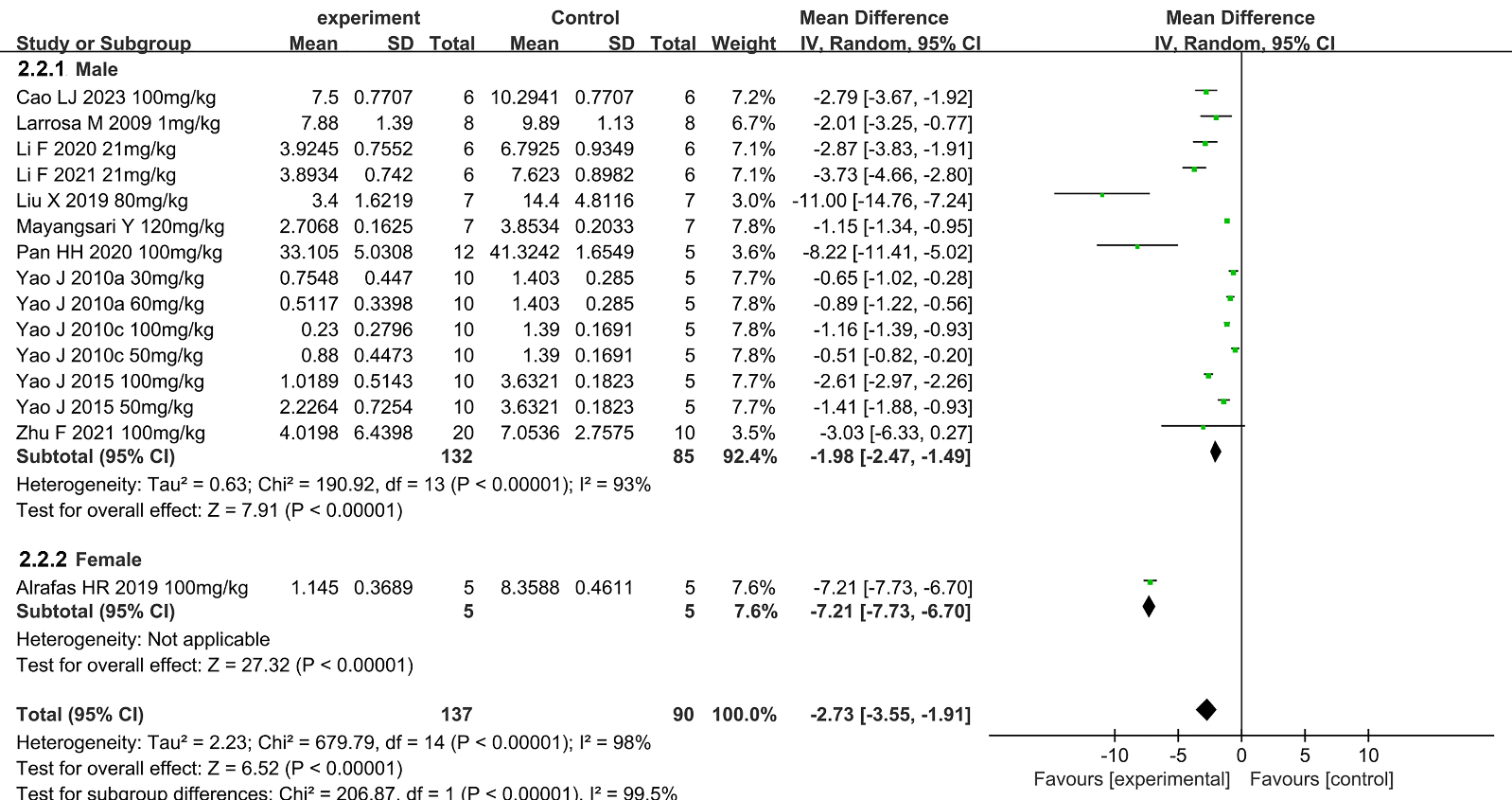


C


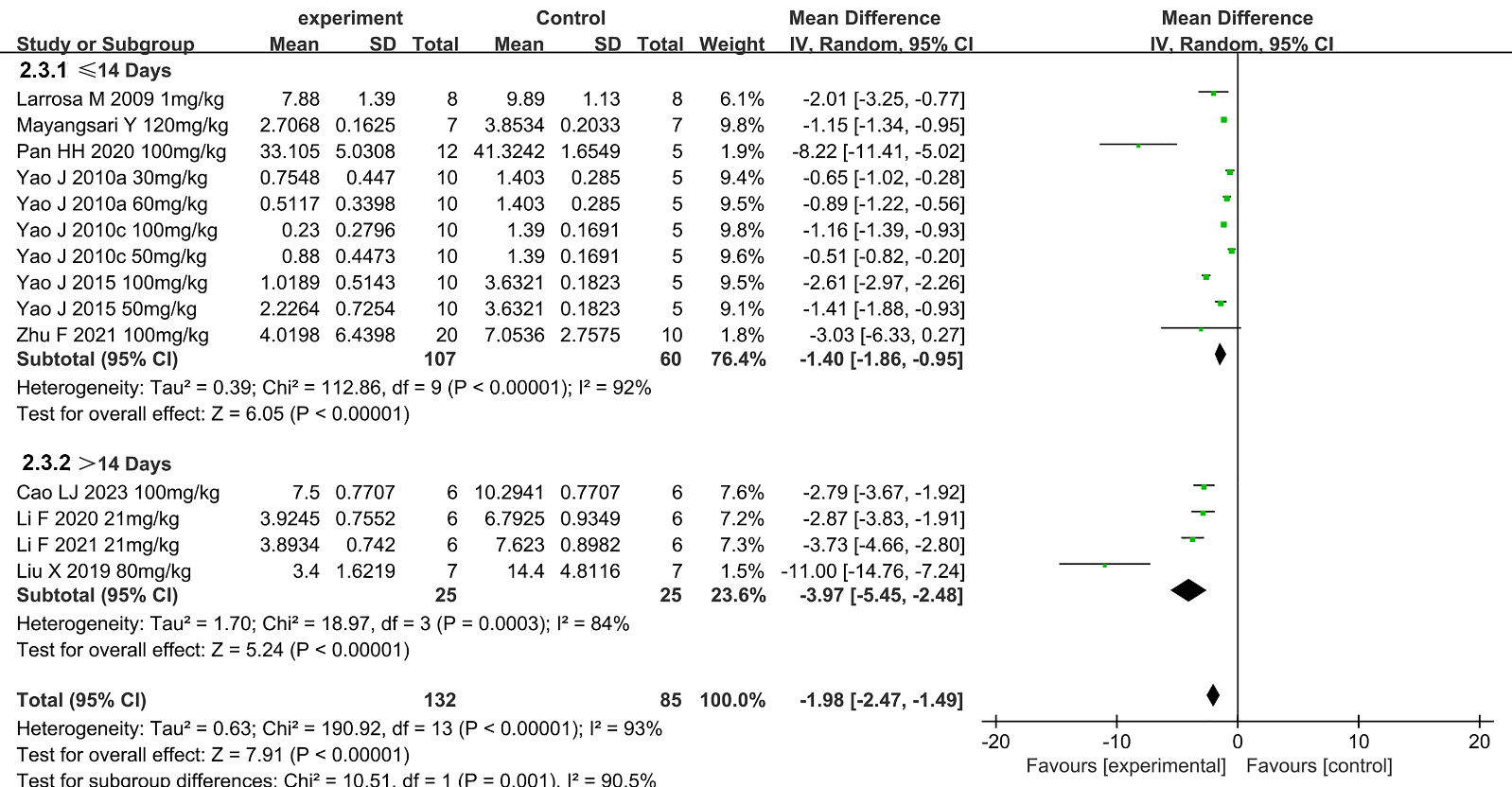


D


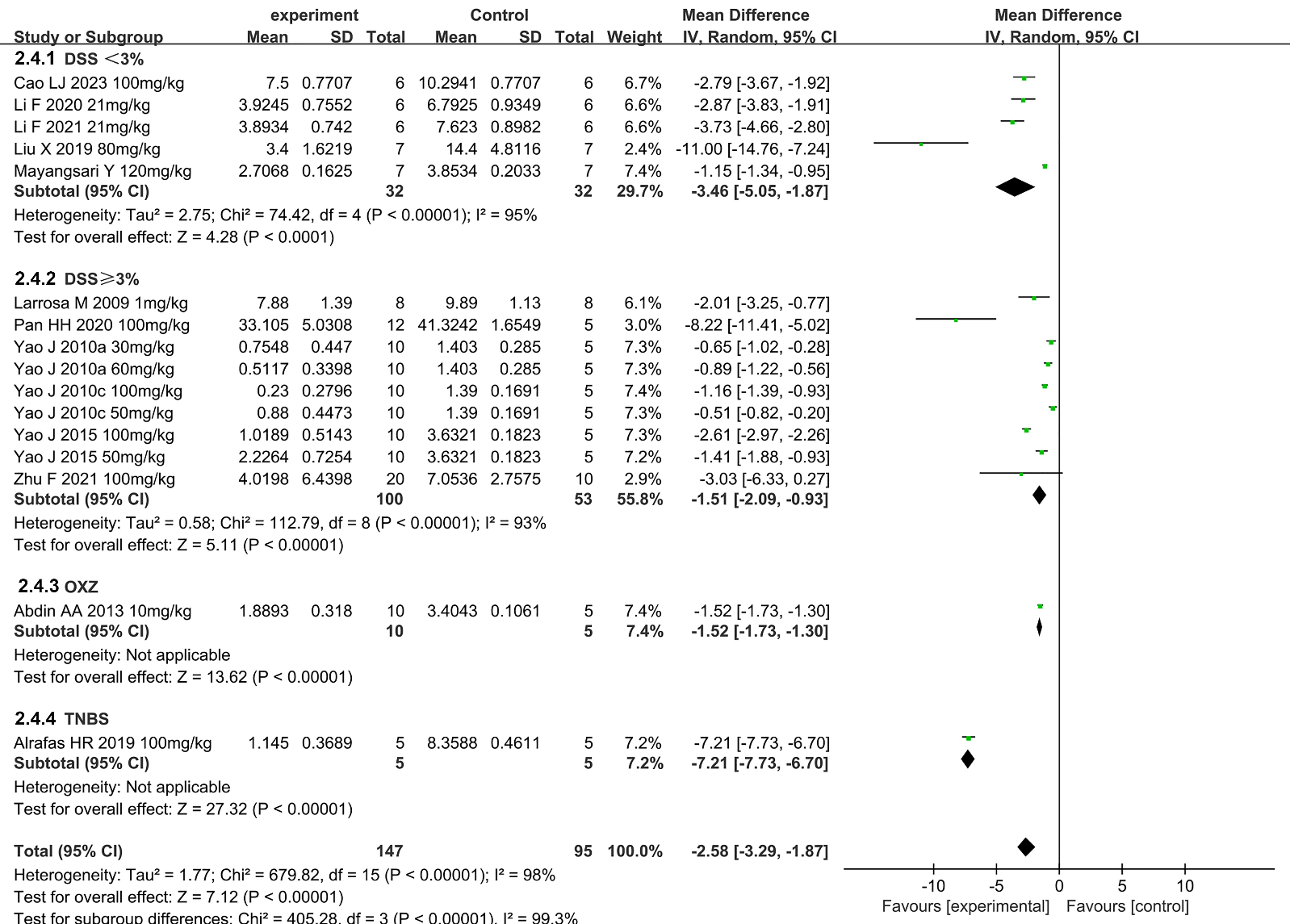


E


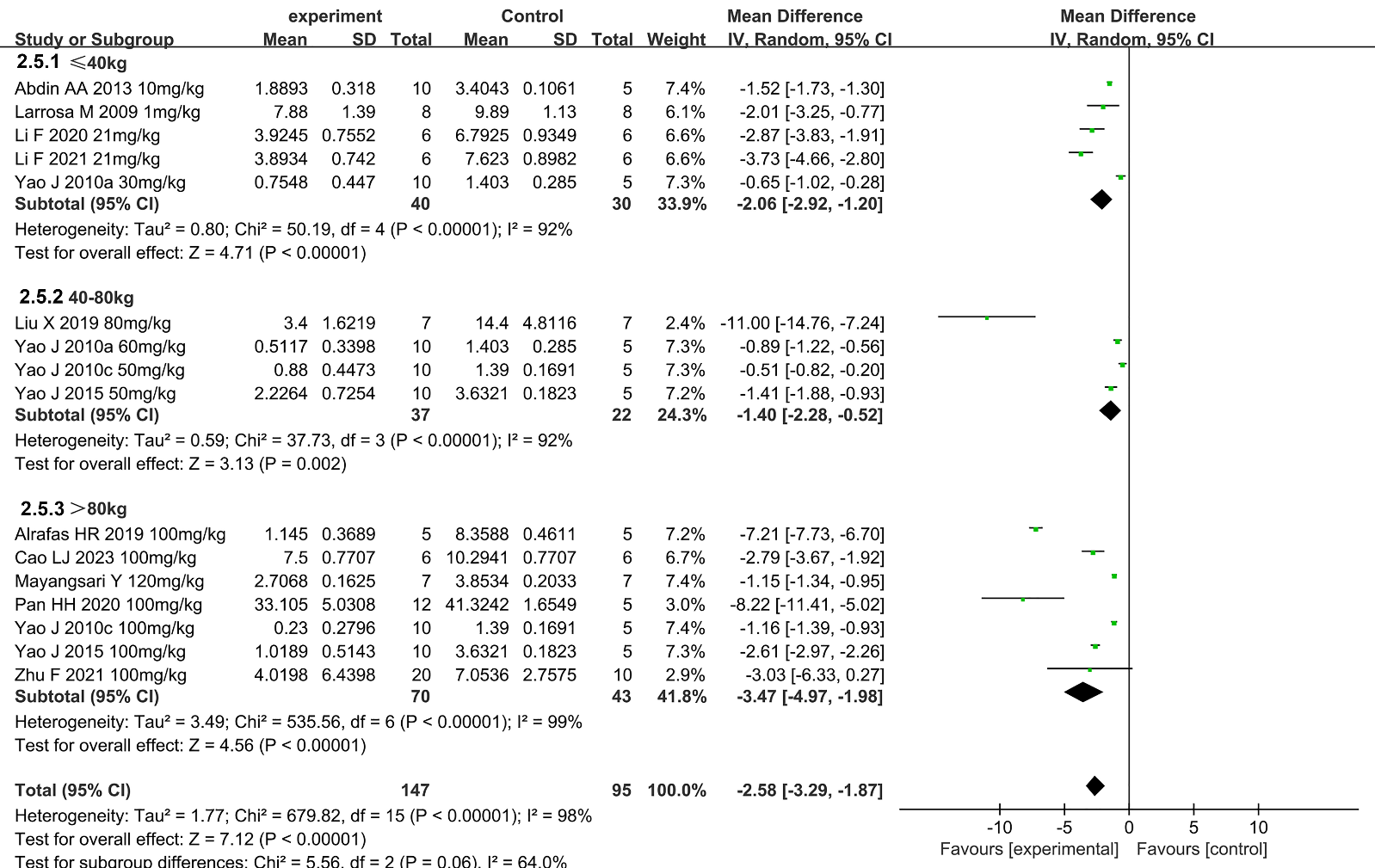


F


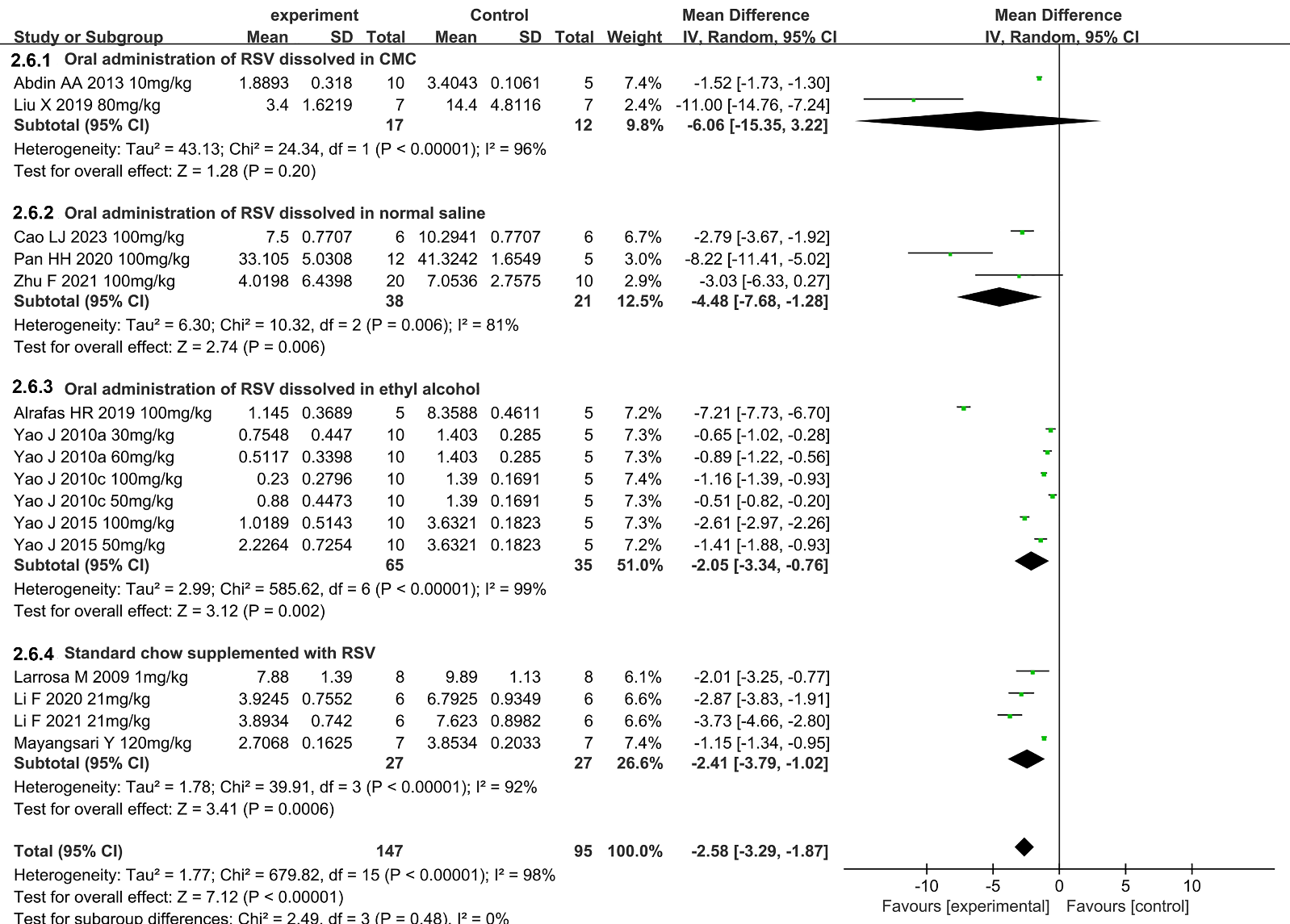


Figure S2. Subgroup analysis of histopathological index. (A) Effect of type of animals on histopathological index; (B) Effect of sex of animals on histopathological index; (C) Effect of the length of molding cycle on histopathological index; (D) Effect of modeling methods on histopathological index; (E) Effect of dosage of resveratrol on histopathological index; (F) Effect of administration methods of resveratrol on histopathological index.

A


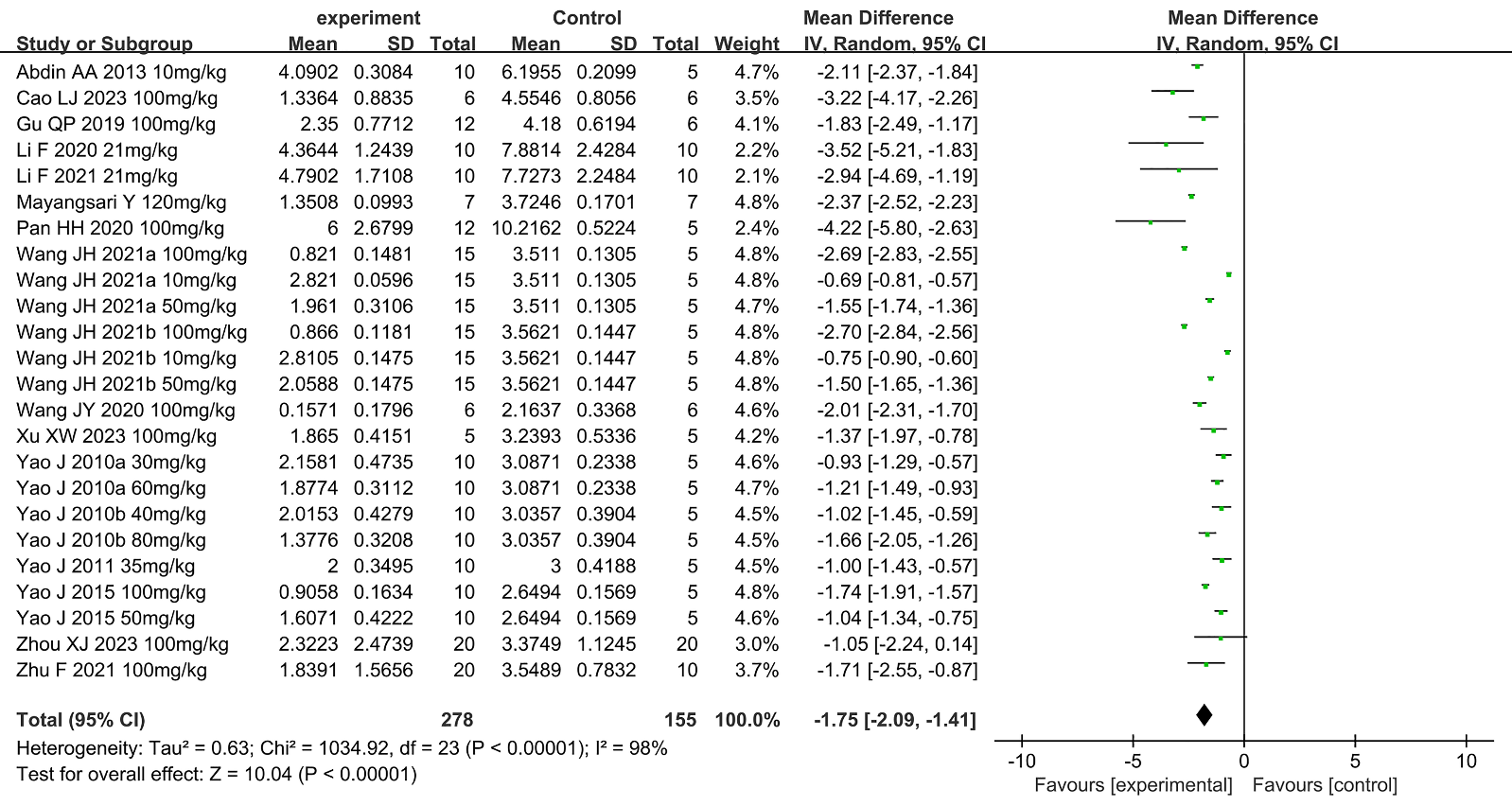


B


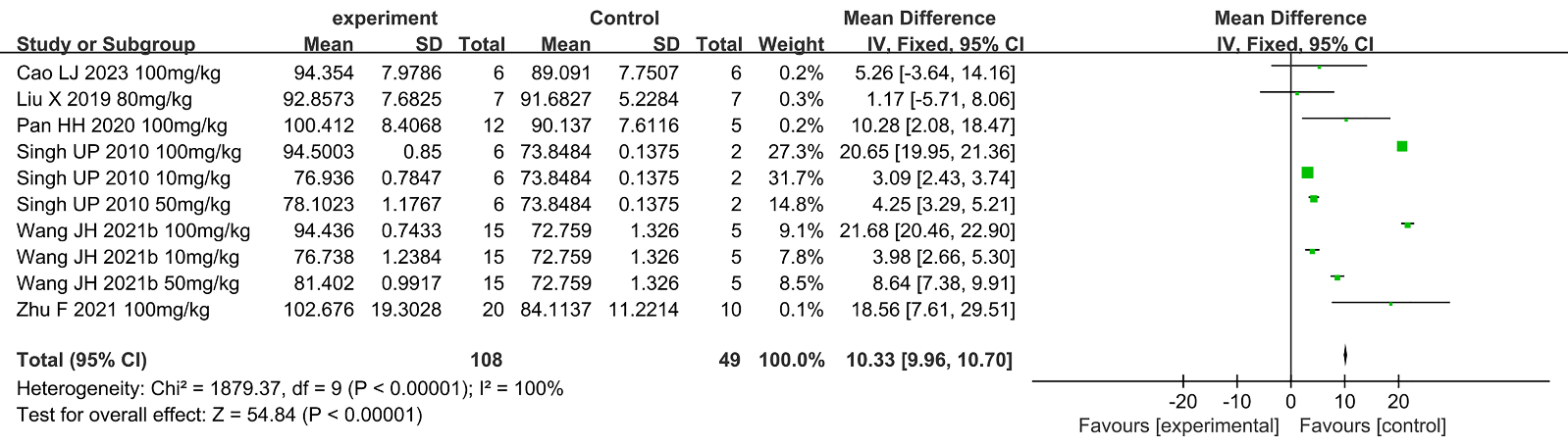


C


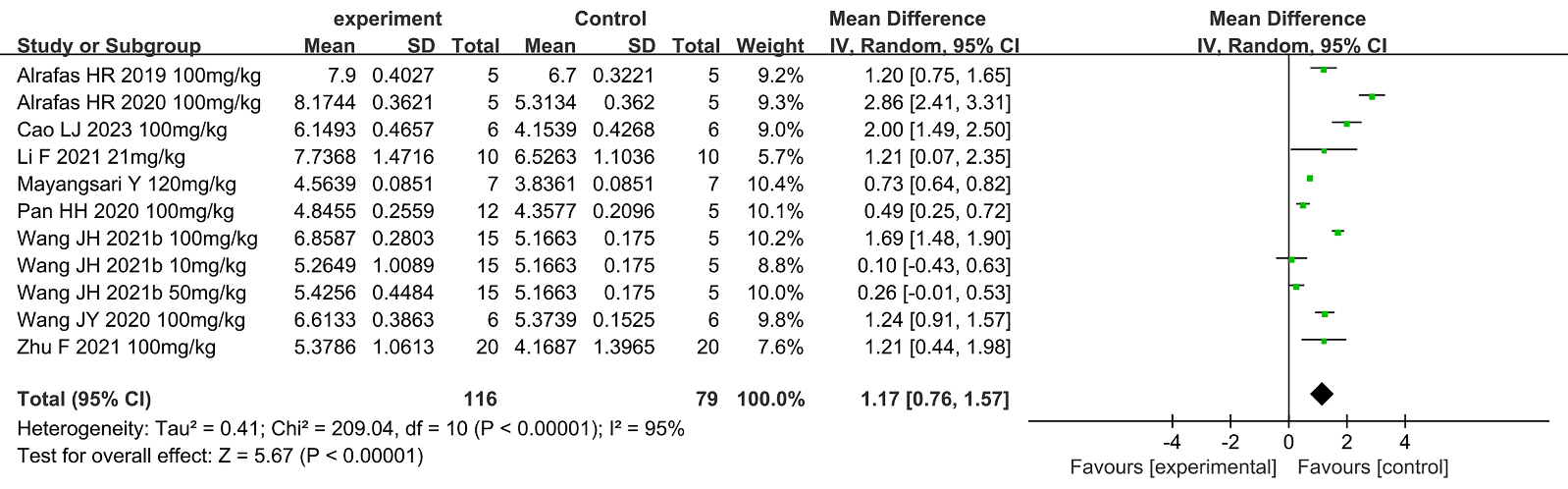


D


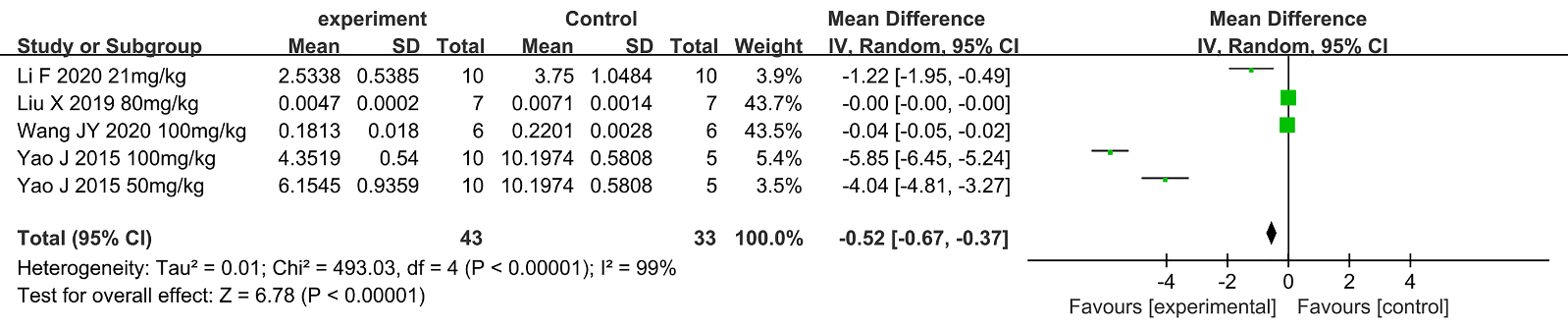


Figure S3. Final DAI score, final weight change and other histopathological indicators. (A) Effect of resveratrol on final DAI score; (B) Effect of resveratrol on final weight change; (C) Effect of resveratrol on colon length; (D) Effect of resveratrol on the spleen index.


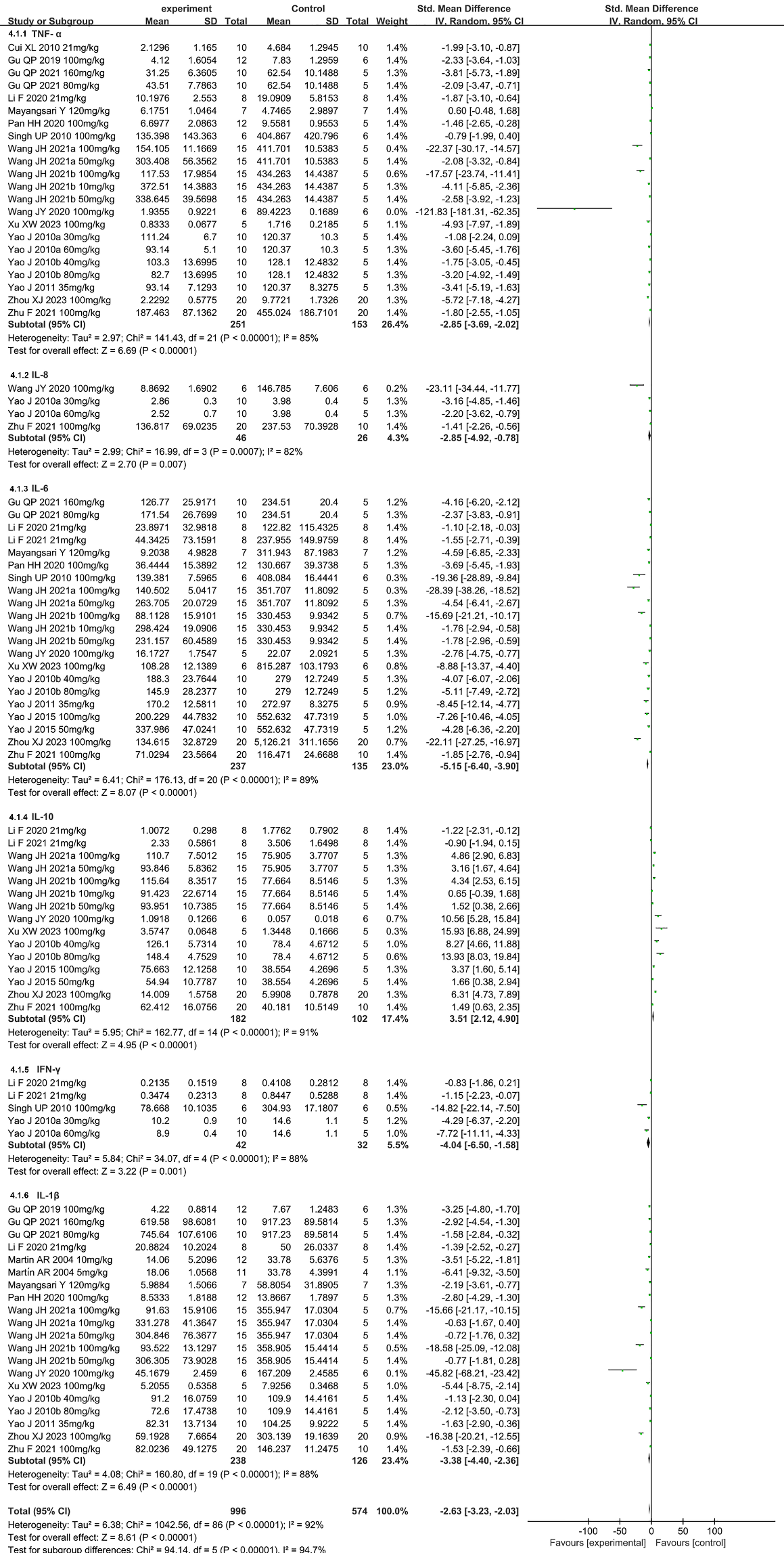


Figure S4. Effect of resveratrol on inflammatory indicators.

A


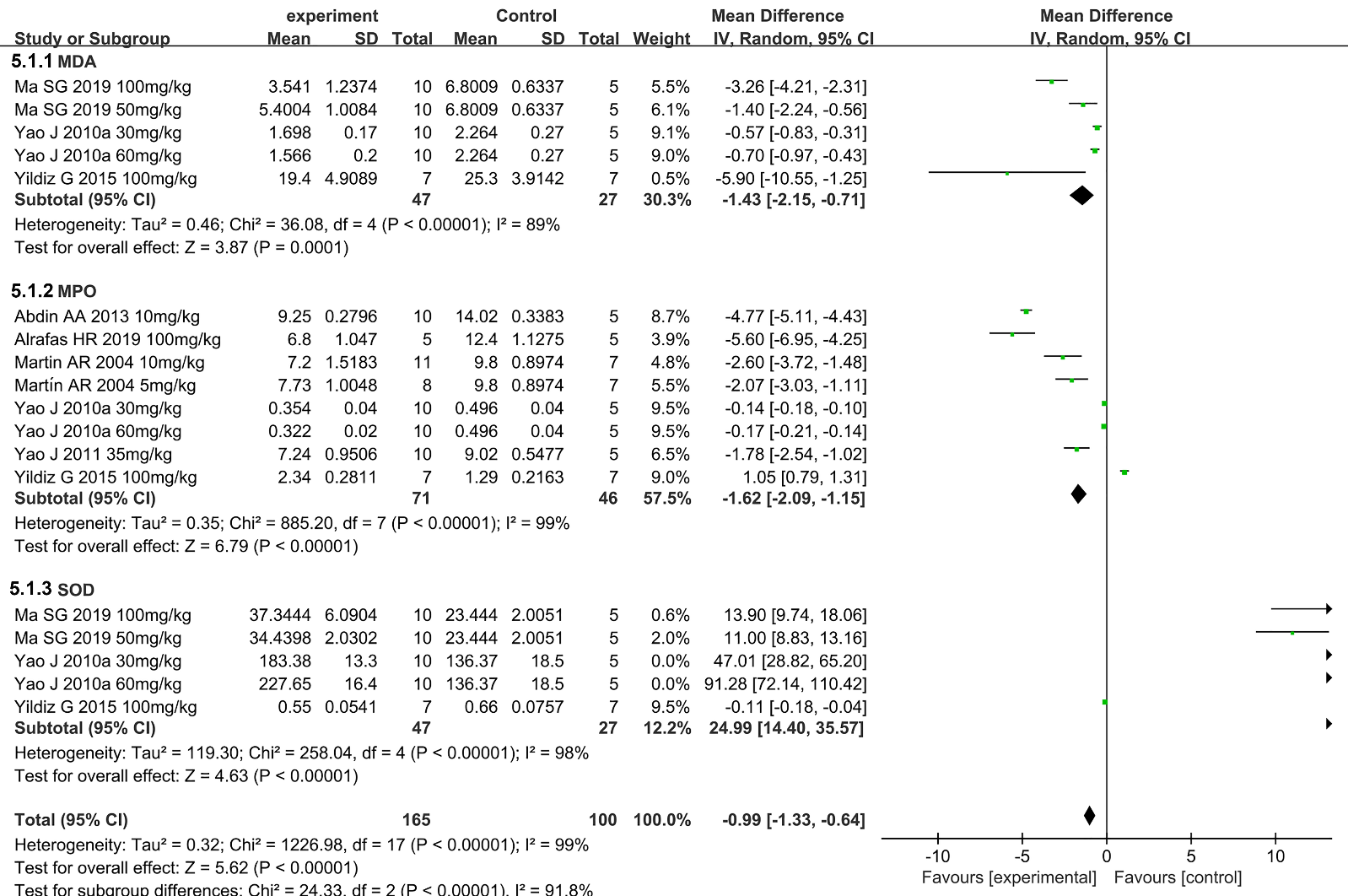


B


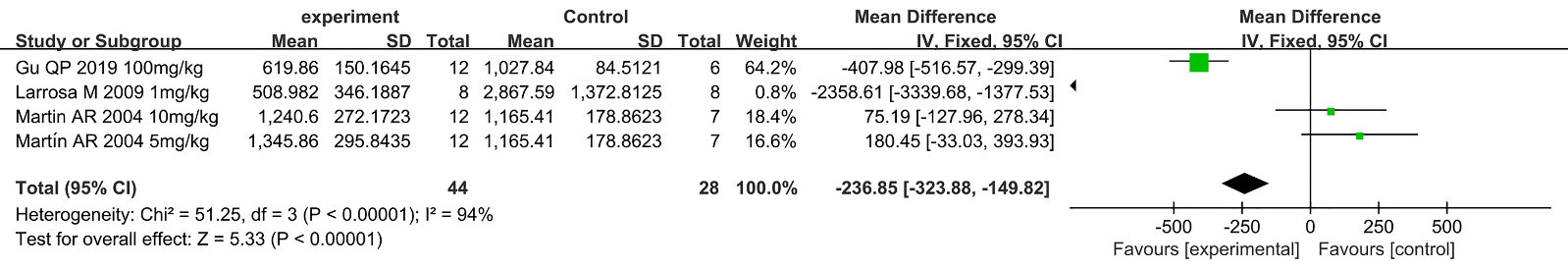


Figure S5. Inflammatory indicators, oxidative stress-related indicators and enzyme metabolites. (A) Effect of resveratrol on oxidative stress-related indicators; (B) Effect of resveratrol on enzyme metabolite (PGE2).

| Table S1. Characteristics of studies excluded based on modeling methods. | | | | | | | | |
| --- | --- | --- | --- | --- | --- | --- | --- | --- |
| **Study** | **Animal** | **Model** | **Sample size** | **Groups** | **Dose** | **Administration method of RSV** | **Assessment time** | **Outcomes** |
| Singh UP 2012 | Female IL-10^-/-^ mice on BL/6 background and BL/6 wild-type  mice aged 10 weeks | Spontaneous formation of a chronic colitis model after 12 weeks without any intervention | 6/6/6/6 | 1. Normal control (BL/6 mice) 2. control   (BL/6 mice + RSV)   1. Model control (IL-10^-/-^ mice + Vehicle) 2. treatment   (IL-10^-/-^ mice + RSV) | 100mg/kg | RSV is orally administered every two days, starting from week 12 and continuing until week 28. | Evaluate weight twice a week | Body weight, SAA, total fecal IgG and IgA, TNF-α, IL-1β, IL-6, IL-12, IFN-γ, RANTES, CD11b^+^ Gr-1^+^ MDSCs (proportion, absolute numbers, and functional characterization), CXCR3 expressing T cells, activated T cells |
| Bilotta S  2022 | Four-week-old male BALB/c wild type mice, or IL-10^-/-^ mice with a BALB/c background | Oral gavage of 50 mg OVA dissolved in 250 µL 0.9% physiologic salt solution (15, 18, 20, 22, 25, and 27d). | OVA enteritis model:  8/8/8/8/8/8 (three for control and three for OVA treatment)  IL-10^-/-^ colitis model: 5/5/5 (BALB/c wildtype mice), 10/10/10 (IL-10^-/-^ mice) | OVA enteritis model:   1. Normal control 2. OVA + water 3. OVA + ethanol 4. OVA + RSV   IL-10^-/-^  colitis model:   1. Normal control 2. IL-10^-/-^ mice + water 3. IL-10^-/-^ mice + ethanol 4. IL-10^-/-^ mice + RSV) | 50 mg/kg | RSV in ethyl alcohol, was gavaged daily for 90 days. | / | OVA:  Mast cell numbers in duodenum and colon tissue, mRNA expression of Mcpt4, Mc-cpa and Il-3rα,  IL-10^-/-^:  Mast cell numbers in duodenum and colon tissue, scores for tissue damage, goblet cell numbers and cell infiltration in colon , survival rate, Degranulation, chemokine and cytokine mRNA expression in BMMC (determine β-hexosaminidase release, Ccl2 and TNF-α ) |
| Sun H  2020 | Male C57/6 mice, aged 8-10 weeks | 7.2 Gy radiation for 5 days | 6/6/6/6/6 | 1. Normal control 2. radiation 3. TBI + vehicle 4. TBI + low-dose resveratrol 5. TBI + medium-dose resveratrol 6. TBI + high dose resveratrol | 50mg/kg,  100mg/kg,200mg/kg | Resveratrol dissolved in 0.1% CMC, was gavaged every day for 7 days before irradiation and then 14 days after irradiation. | Body weight was measured for 13 days (from d1-13) | Body weight, HE staining, IL-1β, TNF-α, NLRP-3, spleen index, thymus index, Sirt1 |

SAA, Serumamyloid A; RANTES, Regulated on Activation, Normal T-Cell Expressed and Secreted; CD11b^+^ Gr-1^+^ MDSCs, CD11b^+^ Gr-1^+^ myeloid derived suppressor cells; CXCR3, Recombinant Chemokine C-X-C-Motif Receptor 3; OVA, ovalbumin; BMMC, mouse bone marrow-derived mast cells; Ccl2, Chemokine ligand 2; TNF-α, Tumor Necrosis Factor-α; TBI, total body irradiation; CMC, carboxymethyl cellulose sodium; IL-1β, Interleukin-1β; Sirt1, Sirtuin 1.
